# Dynamic metabolic modelling of overproduced protein secretion in *Streptomyces lividans* using adaptive DFBA

**DOI:** 10.1186/s12866-019-1591-7

**Published:** 2019-10-26

**Authors:** Jósé R. Valverde, Sonia Gullón, Clara A. García-Herrero, Iván Campoy, Rafael P. Mellado

**Affiliations:** 1 Scientific Computing Service, Centro Nacional de Biotecnología (CNB-CSIC), c/Darwin 3, 28049 Madrid, Spain; 2 Departamento de Biotecnología Microbiana, Centro Nacional de Biotecnología (CNB-CSIC), c/Darwin, 3, 28049 Madrid, Spain

**Keywords:** GSMN, DFBA, Metabolomics, Secretion, *Streptomyces lividans*, Biotechnology, Sec, Tat, Protein overproduction

## Abstract

**Background:**

*Streptomyces lividans* is an appealing host for the production of proteins of biotechnological interest due to its relaxed exogenous DNA restriction system and its ability to secrete proteins directly to the medium through the major Sec or the minor Tat routes. Often, protein secretion displays non-uniform time-dependent patterns. Understanding the associated metabolic changes is a crucial step to engineer protein production. Dynamic Flux Balance Analysis (DFBA) allows the study of the interactions between a modelled organism and its environment over time. Existing methods allow the specification of initial model and environment conditions, but do not allow introducing arbitrary modifications in the course of the simulation. Living organisms, however, display unexpected adaptive metabolic behaviours in response to unpredictable changes in their environment. Engineering the secretion of products of biotechnological interest has systematically proven especially difficult to model using DFBA. Accurate time-dependent modelling of complex and/or arbitrary, adaptive metabolic processes demands an extended approach to DFBA.

**Results:**

In this work, we introduce Adaptive DFBA, a novel, versatile simulation approach that permits inclusion of changes in the organism or the environment at any time in the simulation, either arbitrary or interactively responsive to environmental changes. This approach extends traditional DFBA to allow steering arbitrarily complex simulations of metabolic dynamics. When applied to Sec- or Tat-dependent secretion of overproduced proteins in *S. lividans*, Adaptive DFBA can overcome the limitations of traditional DFBA to reproduce experimental data on plasmid-free, plasmid bearing and secretory protein overproducing *S. lividans* TK24, and can yield useful insights on the behaviour of systems with limited experimental knowledge such as agarase or amylase overproduction in *S. lividans* TK21.

**Conclusions:**

Adaptive DFBA has allowed us to overcome DFBA limitations and to generate more accurate models of the metabolism during the overproduction of secretory proteins in *S. lividans*, improving our understanding of the underlying processes. Adaptive DFBA is versatile enough to permit dynamical metabolic simulations of arbitrarily complex biotechnological processes.

## Background

*Streptomycetes* possess distinctive characteristics that make them an appealing model system for engineering overproduction of biotechnologically interesting products: they are capable of producing a large array of antibiotics and other compounds of interest, and interestingly, large amounts of extracellular proteins [1]. This is coupled with a relaxed DNA restriction system, which facilitates employment of functional plasmids and cloning and propagating heterologous DNA sequences [1, 2]. In addition, *S. lividans* has been shown to efficiently secrete overexpressed proteins [1, 3]. Taken together, these properties make it an attractive host for overproduction of heterologous proteins.

Protein secretion in *S. lividans* takes place through two main routes: the major Sec route which exports unfolded proteins in an ATP-dependent manner and the minor Tat route which secretes folded proteins and seemingly depends on a proton gradient or ΔpH [4] without a consensus cost. Relevant differences have been identified in the cellular response to Sec- and Tat-dependent protein secretion, as well as in the secretion patterns and preference for growth phases [5, 6].

The recent availability of a good quality Genome-scale Metabolic Network (GSMN) model [6] suitable for *S. lividans* 66 and its derivatives *S. lividans* TK21 and *S. lividans* TK24 offers the opportunity to address in-depth studies of the dynamic behaviour of metabolism during protein overproduction and secretion in *S. lividans.* Flux Balance Analysis (FBA) and Dynamic FBA (DFBA) have become a mandatory part of the metabolic analysis toolbox since their introduction [5, 7, 8]. Several implementations are available for carrying out DFBA analyses, each of them with its own advantages and shortcomings [9].

There are, however, situations where the DFBA approach falls short of producing the desired results, leading to the introduction of novel formulations, such as TEAM [10], which can consider the effect of gene expression. Other situations where DFBA may fail to produce accurate results include modelling of initial growth lag; interactive or dynamic modifications of the simulation course, such as the addition of nutrients at specific times (typical of fed-batch cultures), in arbitrary quantities, whenever their concentrations fall below a specific level, or at arbitrary times (as might be done in a laboratory or production environment); the regulated expression of specific routes in response to environmental (e.g. activation of the stress response), or growth-related changes (e.g. activation or inhibition of protein secretion in specific growth phases), simulation of time-dependent system changes (e.g. light-darkness cycles), concentration-dependent boundary conditions, and more. Despite these limitations, little changes have been made to DFBA since its original publication. As a consequence, a number of strategies have been explored to apply flux balance studies, such as limiting analyses to static FBA, running piece-wise simulations, testing alternative optimization problems, coupling secretion to glucose uptake, or forcing switches dependent on nutrient concentration [11–18] among many others.

DFBA calculations may typically be approached using the analytical Dynamic Optimization Approach (DOA) or the stepwise Static Optimization Approach (SOA) [19]. The DOA approach relies on previous knowledge of the simulation conditions to transform DFBA into a non-linear programming model that is solved only once, and thus, is not suitable for the arbitrary introduction of system changes at unpredictable points in time since constraints must be specified beforehand.

This makes SOA better suited to model arbitrary conditions for several reasons: it is scalable to larger metabolic networks, allows use of an instantaneous objective function which gives better results than an endpoint objective function [19], and interactive intervention at arbitrary simulation points is easier to implement permitting total automation of complex simulations.

A new, expanded implementation of DFBA should provide additional versatility to address its major shortcomings: it should facilitate carrying out extended calculations, accommodate arbitrary system changes, either dependent on the environment (e.g. conditional or unconditional nutrient replenishment or removal, such as feed-back driven fed-batch systems) or inherent to the modelled system (e.g. suboptimal strategies, harmonized regulatory responses such as stress reactions or light-darkness cycles), and situations that might violate base DFBA assumptions (e.g. delayed instead of instantaneous or quasi-instantaneous responses). These additions would allow dynamic metabolic modelling of previously untreatable yet biologically or industrially interesting systems.

Here, we introduce an Adaptive DFBA algorithm that allows calculation of arbitrarily complex simulations, apply for the first time advanced statistical methods to mine relevant data from simulation results and to explore the construction of predictive models of the evolution of metabolic fluxes during overproduction of secretory proteins in *S. lividans*.

## Implementation

We have chosen to implement our approach using the R-based, fully open source sybil system [20], starting from the sybilDynFBA package. Sybil was chosen over OpenCOBRA [21] because it has proven more computer-efficient in our tests, does not require a MATLAB™ license and follows very closely the well-known COBRA implementation of DFBA. We use the SOA approach [5, 7, 8, 19] to solve the time-dependent flux balance problem in our simulation. At each time step in the SOA simulation, we have implemented the solution of the flux problem using alternative FBA solvers from sybil. We also provide the choice of adding a Minimization of the Total Flux (MTF) calculation after FBA at each step to reduce the impact of multiple optimal solutions in FBA [22].

Solution of the system of ordinary differential equations (ODE) relies on a library to solve the ODE problem. We have tested the GLPK [23], CLP [24], LP-SOLVE [25] and CPLEX [26] libraries with our code in various systems and hardware architectures.

The method has been tested using known data on the metabolism of *S. lividans*, either plasmid-free, pIJ486 plasmid-bearing, or over-expressing and secreting Tat- and Sec- dependent overexpressed secretory proteins, and comparing the results with published data [3, 27–31].

All programming has been done using R and is publicly available on GitHub (see Additional file 1: Table S1 for sample commands to reproduce our results).

### Overall description of the algorithm

The Adaptive DFBA algorithm is loosely derived from pre-existing code. The R implementation, sybilDynFBA is itself a derivative from dynamicFBA in OpenCOBRA which it aims to reproduce as closely as possible. Inspection of both codes allowed us to identify their assumptions and implicit limitations, which have been removed to the extent possible in Adaptive DFBA. The details of the improved algorithm are described below, emphasizing its differences with previous implementations.

#### Pre-processing step

In this step, all input data is validated for proper typing and validity, values are revised and, data is prepared for the main computation loop:

##### Biomass reaction

Traditional DFBA code assumed that the objective function was Biomass. In Adaptive DFBA, Biomass calculation is decoupled from the objective function, ensuring proper calculations under unrelated, uncorrelated or competing objectives, and allowing use of multiple objectives. The biomass reaction may be specified as an argument, but if it is not, the model will be inspected to find a suitable one, only if this fails the first objective function will be used after issuing a warning.

##### Exchange reactions

Adaptive DFBA removes the requirement to specify substrates and concentrations in the same order they are defined in the model, which was implicit in former implementations.

The classical codes assumed that metabolites with zero concentration were available in excess, thus, absent substrates could only be modelled by using an initial trace concentration. Adaptive DFBA allows initial zero concentrations to specify absent substrates, and excess substrates are indicated by negative concentrations.

##### Uptake limits

During an Adaptive DFBA simulation, reaction limits may be arbitrarily changed, potentially leading to an inconsistent state. The initial uptake limits are checked and saved to allow recovering the original state when needed.

##### Final initialization

All variables and data structures needed for the simulation are initialized, and a progress bar (text or graphic depending on verbosity level) is prepared.

#### Main loop

At each step required system modifications are applied before calculation. Modifications are divided in two classes:

##### Changes to model status

Any reaction limit can be modified at any time point using one of two forms:

1. A data frame containing as many rows as time points and where each column will correspond to a reaction limit; each limit is identified by its name followed by ‘[upp]’ (upper limit) or ‘[low]’ (lower limit). Only reactions whose limits are to be changed need to be listed. Values must be specified at every time point.
  Since under experimental conditions exchange rates are typically measured only at specific time points, we considered the possibility of specifying only these values and providing various interpolation mechanisms. However, since implementing any imputation scheme is easy in R, we considered best to leave interpolation to the user.
2. A function that takes as arguments the model, the current concentrations, the last previously computed fluxes and the time step, and which returns a new model. The function may use these values to implement an imputation/interpolation scheme as in the matrix approach, as well as interactive changes in response to the environment

##### Changes in metabolite concentration

We assume that this will be used typically to model external interventions on the system, such as predictable changes (like progressive increment or exhaustion of a metabolite by external actions or addition/removal of metabolites at key time points) and feed-back controlled actions in response to metabolite changes (such as a sensor/actor system in a controlled fermentation that injects or depletes nutrients when concentrations cross a given threshold). Again, there are two possibilities:

1. A data frame of predefined changes: a table of changes to apply at each time point to the substrate concentration. Since it works as a differential engine, a zero means no change to current concentrations, a positive value an addition and a negative value a removal of metabolites.
2. A function that will take as input the current concentrations and return the new concentrations to be used. Using a function provides the additional control needed to implement feed-back intervention in response to dynamic system changes.

##### Time-step derived constraints

At any time point, the concentration of each metabolite is checked against the substrate uptake rate to avoid overusing non-existing resources. In the original DFBA algorithm, the direction of each reaction was predefined by the initial limits. This is sensible when nutrients are added only at the onset and biomass can only grow, but is unsatisfactory when any concentration or exchange limit can change at any time during the simulation or a metabolite is excreted and later consumed (and vice versa). In Adaptive DFBA, the lower rate of an exchange reaction is modified to avoid over-consumption only if the reaction is explicitly an uptake reaction at the current time step.

##### Solution of the system of equations

The classic DFBA algorithm uses FBA to solve the metabolic problem at each step. The result of FBA is not uniquely defined [5, 7, 8]. We have checked alternative FBA implementations: in these cases, although the solutions produced may diverge at specific time points, they tend to return to common solutions in later steps, leading to practically equivalent dynamic trajectories (data not shown).

Solution of the FBA system of equations relies on an auxiliary ODE solver library. We checked GLPK, CLP, LP-SOLVE and CPLEX on different computer architectures. All of them should produce the same or approximately similar (within rounding error bounds) results. However, in our experience, we found that LP-SOLVE gave different results in several of the calculated simulations, GLPK failed to run in only one specific combination of simulation parameters, operating system and architecture (whose origin we could not track), CLP worked correctly in all cases but was significantly slower than GLPK, and CPLEX was tested only in the free, academic version, which accepts a limited number of equations: for systems within this limit, it was the fastest method, but we could not check it with larger systems.

Uncertainty in FBA may be reduced using optimal fluxes calculated through Minimization of the Total Flux (MTF), leading to a hopefully more parsimonious solution. We have implemented MTF as an optional alternative to FBA, and found that it leads to a system evolution that is practically equivalent to that obtained by FBA. Since MTF computation time is larger, FBA has been kept as the default option, and MTF is offered on demand.

Both, FBA and MTF compute a single value for each metabolic flux at each step. It would be more useful to obtain an estimate of flux variability limits. We have tested as well implementing Flux Variability Analysis (FVA) calculations at each time step to obtain stepwise flux limits for each reaction. FVA results in a huge increase in computational time that would only be justified under special situations. We are currently exploring the best approaches to definitively integrate FVA within the algorithm.

##### System update

After the system has been solved, biomass content is calculated using the actual Biomass reaction instead of the objective function. This allows study of the evolution of a system using an objective other than (and possibly conflicting with) Biomass. The Biomass calculated is combined with the computed fluxes to calculate new concentrations for the next step as in the traditional scheme.

#### Output and post-processing

We have ensured that the return of Adaptive DFBA remains fully compatible (and thus can substitute it seamlessly) with DFBA, easing its integration in existing simulation schemes. Additional information can be obtained using increasing verbosity levels.

### Statistical analysis

#### Correlation/regression tests

During simulations, both fluxes and metabolite concentrations computed at each step were collected for further statistical analysis, their distributions were checked for normality using the Anderson-Darling and Shapiro-Wilk tests, prior to selection of subsequent analyses. Correlation and regression analyses were used to study the relationship between all possible pairs of same-type variable distributions: we considered the Pearson product-moment, Spearman Rank and curvilinear regression tests, which were applied to all possible pairs of variables. Curvilinear regression was used to fit quadratic and cubic polynomial equations.

#### Feature extraction and variable importance

To identify which variables may have a stronger influence on desired outcome variables, we built models where each desired outcome was initially modelled from all existing variables, and used three feature extraction methods to select the most significant contributors: forward selection, backward selection and Boruta’s algorithm (a machine-learning/artificial intelligence algorithm based on Random Forest Classification) were applied to determine the relative variable importance in explaining each outcome sought. For the application of Boruta’s algorithm, variables were divided in groups of 20, repeatedly applying selection and regrouping until there remained 20 or less significant variables which were subject to a last selection iteration. At each step, critical judgement based on metabolic understanding was used to supervise automatic selection.

#### Comparison of time-dependent metabolic distributions

Similarities and differences in time-dependent distributions were studied comparing either whole or partial timelines. Group comparisons were carried out using ANOVA and Kruskal-Wallis, followed by Welch modification of Student’s t-test or the Mann-Whitney/Wilcoxon test. Metabolites whose evolution was clearly different were selected to build time-dependent predictive models that might be used to differentially steer simulations, fitting their time-dependent evolution to either linear, quadratic or cubic models. The parameters for the resulting models were then compared with the experimental data using two non-nested model comparison methods, the Cox test and the David-Mackinnon J test.

## Results

We have exploited Adaptive DFBA to model heterologous protein overproduction and secretion in *Streptomyces lividans,* in the presence of conflicting and uncorrelated objectives.

For simplicity, in all subsequent simulations, we have used the following scheme: system interventions have been reduced to the minimum, time step was set at 1 h, constraints were defined only at a few key times (typically every 12 h or more, punctually using a 4–6 h time point to soften growth phase transition), and values at intermediate points were imputed using linear interpolation, corresponding to multi-phase linear regression [27, 31]. Despite these settings, the resulting simulations were remarkably well-behaved.

### Descriptive modelling

The systems modelled correspond to *Streptomyces lividans* TK24 strains growing on a complex medium (NMMP, supplemented with glucose and casamino acids). The experimental data has been originally published elsewhere as figures [27].

#### Wild-type Streptomyces lividans TK24

DFBA and Adpative DFBA models are illustrated in Additional file 1: Figure S1. The DFBA model predicts the cross-over of NH_4_^+^ with glucose, glucose exhaustion and some amino acid uptake rates evolve similarly to the experimental observations, however, other rates such as growth arrest and L-alanine excretion and later uptake are not reproduced.

To properly model growth and L-alanine exchange, they had to be steered during the simulation using Adaptive DFBA, which permitted forcing early excretion of L-alanine and releasing this constraint later in the simulation.

#### *S. lividans* TK24 strain harbouring the multi-copy plasmid pIJ486

In the simulation of *S. lividans* TK24 harbouring the multi-copy plasmid pIJ486 (model *S.lividans-*TK24-pIJ486), the plasmid imposes a strain that can be detected as a growth delay (Additional file 1: Figure S2). With DFBA, besides the previous limitations, and since growth and C source exchange rates can only be set initially to fixed rates, the system consumes and exhausts glucose too fast.

Adapting glucose uptake to its concentration in the medium using Adaptive DFBA allowed simulation of gene-regulated adaptation to nutrient depletion, making it available for a longer time. Additionally, at low levels of glucose uptake, an alternate C source is needed, requiring L-alanine to be switched from excretion to uptake: only controlling both switches could growth be maintained. Use of L-alanine resulted in a corresponding reduction in NH_4_^+^ needs, which automatically started being excreted. The observed shoulder in growth rate matched a less-defined region (identified by a larger dispersion) in published growth curves [27, 28].

#### *S. lividans* TK24 pIJ486 overproducing sec-secreted mTNF-α

**Fig. 1:**
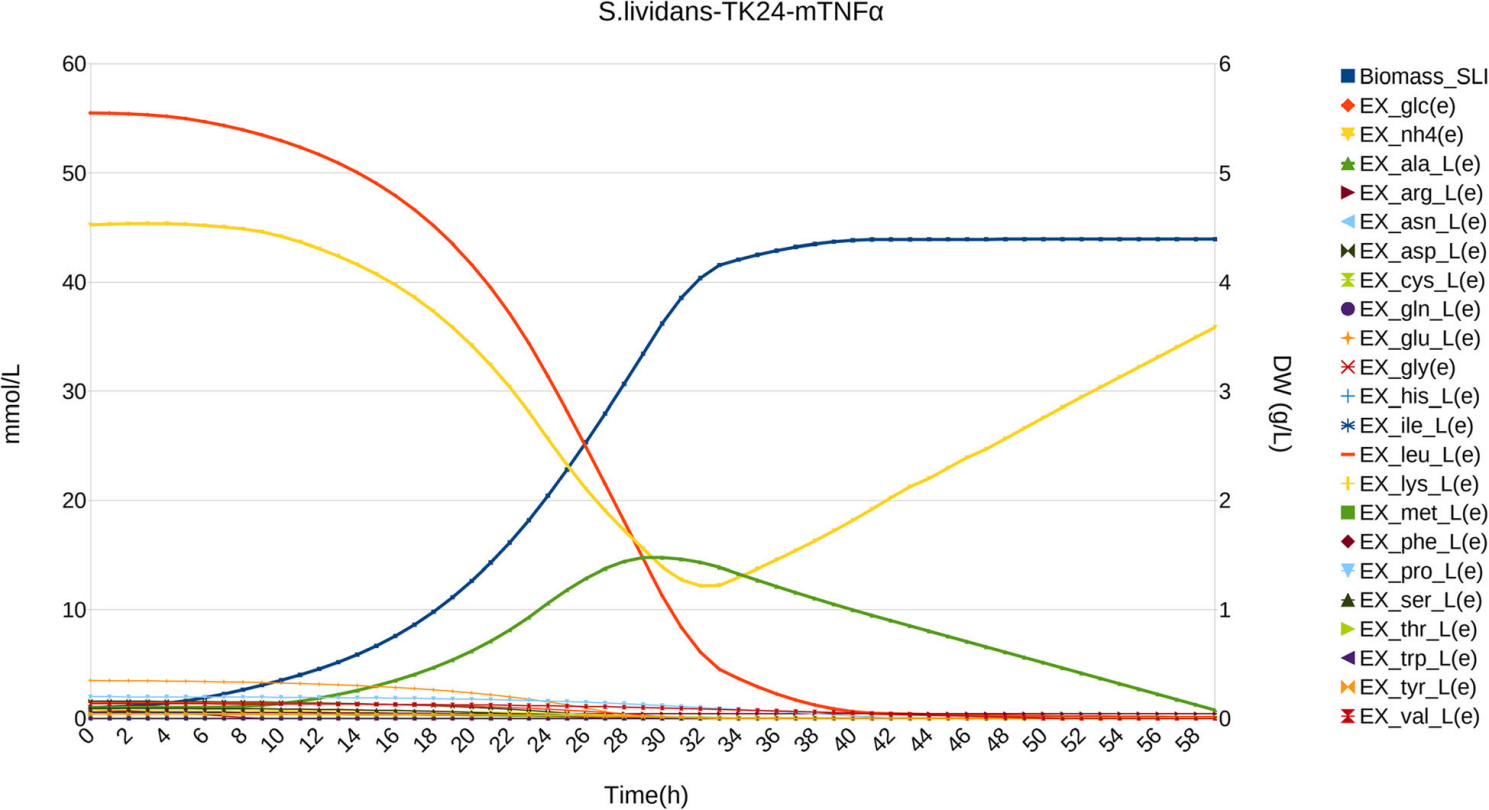
Simulation of *S. lividans* TK24 pIJ486 overproducing Sec-secreted mTNF-α. Biomass is expressed as dry weight (DW) in g/L, time is expressed in h, and metabolite concentration in mmol/L. For simplicity, only glucose, NH_4_^+^ (dotted lines) and amino acids have been plotted, and Biomass and L-alanine have been emphasized using special symbol marks, as described in the legend

Simulation results for *S. lividans* TK24 pIJ486 overproducing Sec-secreted mTNF-α (model *S.lividans-*TK24-mTNFα) are presented in Fig. 1 and Additional file 1: Figure S3. Protein secretion of mTNF-α is associated to growth rate except in late phases [27]. This should make it susceptible to modelling using traditional DFBA. However, like in previous simulations, DFBA produces incorrect results (Additional file 1: Figure S3A).

To match experimental rates (Fig. 1) we had to consider the modifications learnt from previous simulations with Adaptive DFBA. Still, the initial simulations revealed a nutrient imbalance if the original protein secretion rate was maintained through later stages (as in traditional DFBA). This nutrient imbalance could not be satisfied with the remaining nutrients, and the simulation ended prematurely. Thus, proper simulation also required a progressive time-dependent adjustment of mTNF-α secretion at late times (Fig. 1).

### Exploratory use of adaptive DFBA

We now proceed to show the use of Adaptive DFBA to explore the metabolism of systems with limited knowledge: first, we analyse *S. lividans* TK21 grown in a complex medium (NMMP complemented with mannitol and casamino acids), over-expressing secretory proteins cloned in the multi-copy plasmid pIJ486 and secreting them through the minor Tat or the major Sec secretion routes, using model proteins that have been shown to display secretion patterns clearly dissociated from growth [28, 29]. Previous studies have shown that the strains of *S. lividans* TK21 behave similar to those of *S. lividans* TK24, when grown on similar media [28, 29], and that the metabolic model should be (to the extent known) transferable between them [6].

#### Tat-dependent agarase overproduction in *S. lividans* TK21

Strain *S.lividans* TK21(pAGAs5) was generated transforming *S. lividans* TK21 with the multicopy plasmid pIJ486 carrying the gen *dagA* encoding agarase from *S. coelicolor* and its regulatory region (model *S.lividans*-TK21-DagA)*.* Tat-dependent secretion typically starts by the end of exponential growth, when the cells enter the stationary phase, increasing when nutrients become scarce in the medium. In the case of agarase, there is also some reduced early secretion detectable during the second exponential growth phase [3, 6, 28, 29]. Hence, secretion cannot be coupled to growth nor can it be held constant throughout the simulation. Additionally, agarase secretion has been detected after 60 h of cultivation, secretion takes place at higher yields, and only limited experimental data is available. Hence, the application of exploratory methods is of interest as an expeditive route to inquire in the underlying metabolic processes.

DFBA was unable to produce a proper simulation: besides the previous issues, if the lower limit on protein secretion was set to zero to accommodate early lack of agarase secretion, it was never secreted ignoring any upper limit, as agarase overproduction competes with growth. On the other hand, setting the lower limit to a higher value to force late secretion would also force early production, with an unrealistic initial evolution of the system, and leading to early termination of the simulation due to exhaustion of the C source (Additional file 1: Figure S4A).

**Fig. 2:**
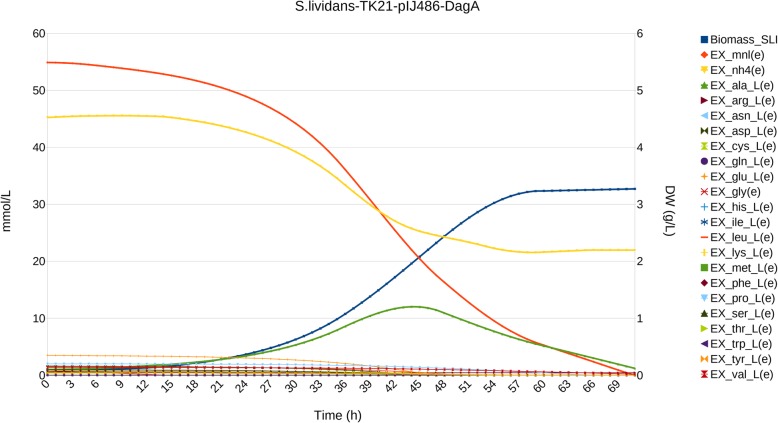
Simulation of *S. lividans* TK21 pIJ486 overproducing Tat-secreted agarase. Biomass is expressed as dry weight (DW) in g/L, time is expressed in h, and metabolite concentration in mmol/L. For simplicity, only glucose, NH_4_^+^ (dotted lines) and amino acids have been plotted, and Biomass and L-alanine have been emphasized using special symbol marks, as described in the legend

Adaptive DFBA allowed us to progressively adapt agarase secretion to match the levels detected in each growth phase, as well as to exploit the experience learnt from previous simulations about other relevant constraints, such as adapting rates in mannitol and L-alanine exchanges (Fig. 2).

Preferential use of amino acids was associated to reduced needs for NH_4_^+^, both at the onset, and in the last phases of the simulation, when L-alanine becomes the preferred C and N source, although now the effect was smaller, reflecting reduced levels of L-alanine and a larger need for N for protein synthesis.

We have also used this model to explore simulation of alternative scenarios, running simulations using a conservative estimate for Tat secretion cost and more aggressive costs considering secretion driven by a proton motive force (PMF) of 80,000 H^+^ or its proposed equivalent of 10,000 ATP [6, 30]. Inspection of the evolution of major nutrients suggests that these alternate costs have a reduced impact, visible at very late stages (after 60 h) of the simulation (data not shown).

#### Sec-secreted α-amylase overproduction by *S. lividans* TK21

Strain *S. lividans* TK21 (pAMI11) [29] was generated using a pIJ486 plasmid derivative carrying the *amlB* gene encoding α-amylase from *S. lividans* under the control of its own promoter (model *S.lividans*-TK21-amlB). Simulation requires considering a secretion pattern clearly different from that of mTN-α: α-amylase is maximally secreted at the exponential phase, and progressively less during the stationary phase until it becomes undetectable, making DFBA constant rates inappropriate (Additional file 1: Figure S5A).

**Fig. 3:**
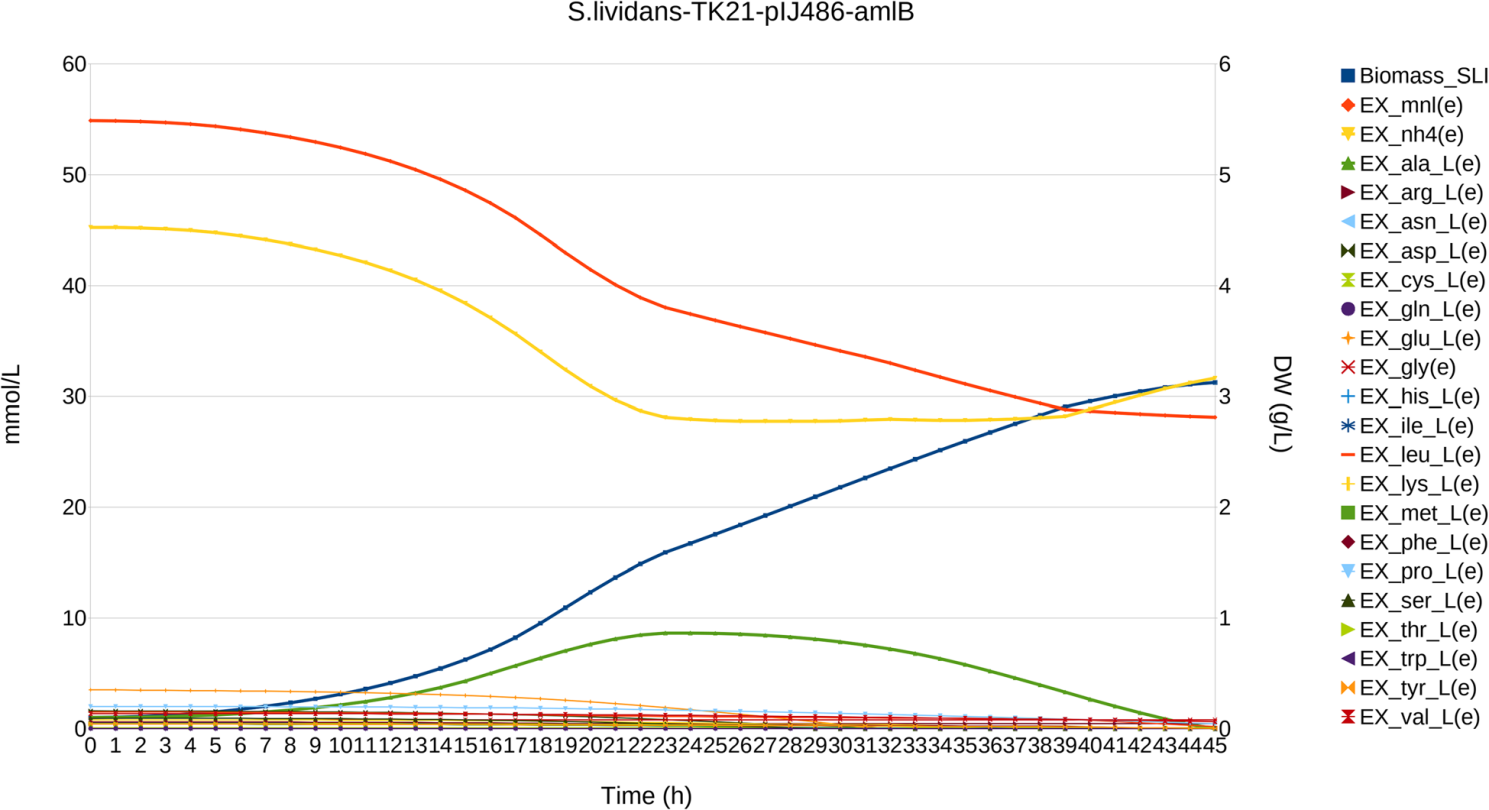
Simulation of *S. lividans* TK21 pIJ486 overproducing Sec-secreted α-amylase. Biomass is expressed as dry weight (DW) in g/L, time is expressed in h, and metabolite concentration in mmol/L. For simplicity, only glucose, NH_4_^+^ (dotted lines) and amino acids have been plotted, and Biomass and L-alanine have been emphasized using special symbol marks, as described in the legend

Using Adaptive DFBA we could take advantage of lessons learnt from previous simulations and, additionally, to completely uncouple secretion from growth simulating the known secretion pattern. In this case, since there is practically no protein secretion requiring additional N consumption, the inverse relationship between NH_4_^+^ and L-alanine was more evident during the stationary phase (Fig. 3).

#### Sec-secreted overproduction of cellulase-a by *S. lividans* TK24 pIJ486

**Fig. 4:**
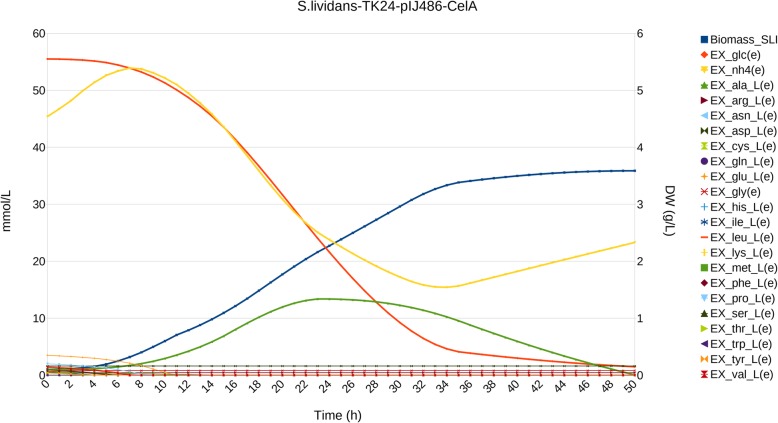
simulation of *S. lividans* TK24 pIJ486 overproducing Sec-secreted cellulase-A. Biomass is expressed as dry weight (DW) in g/L, time is expressed in h, and metabolite concentration in mmol/L. For simplicity, only glucose, NH_4_^+^ (dotted lines) and amino acids have been plotted, and Biomass and L-alanine have been emphasized using special symbol marks, as described in the legend

We have also simulated growth of *S. lividans* TK24 harboring the pIJ486 multicopy plasmid and expressing the *Rhodothermus marinus* thermostable cellullase-A gene *celA*, cloned behind the promoter and signal sequence of *Streptomyces venezuelae* subtilisin inhibitor (*vsi*) (model *S.lividans*-TK24-CelA) grown in a different, C-limited minimal medium containing glucose and casamino acids [31, 32]. We extended our *S. lividans* model to accommodate overproduction and secretion of cellulase-A, and to use the new experimental conditions. As in previous cases, traditional DFBA failed to reproduce experimental observations (Additional file 1: Figure S6A), whereas applying the knowledge gathered from previous simulations allowed us to steer the simulation using Adaptive DFBA to reproduce observed rates (Fig. 4).

Comparison with the simulations of α-amylase and mTNF-α showed that amino acids were exhausted faster. Although barely appreciable, switches on amino acid and NH_4_^+^ consumption were also coupled to small changes in the slope of the growth curve. These effects can only be ascribed to the composition and size of the protein secreted, and suggests that overproduction of cellulase-A imposes a distinct stress on the cell, in agreement with recently published data [32].

### Statistical analysis

The exploratory models built for Tat-dependent agarase secretion and Sec-dependent α-amylase secretion were subjected to statistical analysis to determine dependencies and identify the most relevant variables affecting protein production. Although strong positive/negative correlations (R > |0.9|) between uptake of some amino acids and protein production could be identified, consumption of amino acids is not enough to explain protein production as secretion may be detected after amino acids have been consumed or may be absent in their presence (e.g. Additional file 1: Figure S7 and Additional file 1: Figure S8). Similarly, no other metabolite showed a clear dependence relationship with protein secretion when considered in isolation.

Feature selection was applied to models initially including all metabolites to select the most important variables in the model (e.g. Additional file 1: Figure S9). H_2_O_2_ was consistently among the variables most relevantly associated with agarase and α-amylase secretion. Interestingly, L-alanine, mannitol, other amino acids and four metallic ions (Mo^2+^, Ni^2+^, Cu^2+^, and Co^2+^) are consistently among the most significant variables for both proteins.

Regarding comparison of time-dependent changes, none of the variables was normally distributed (as may reasonably be expected), so only Mann-Whitney U test results were finally considered. Visualization with heat-maps (e.g. Additional file 1: Figure S10) proved the most practical way to explore the comparisons between simulations and to identify likely relevant differences; these were selected for further analysis consisting in the construction of regression models that were subsequently subject to model comparison analyses. Generally speaking, the time-dependent regression models considered resulted only in roughly approximate predictions (e.g. Additional file 1: Figure S11), and the relative success of each model heavily depended on the shape of the original distribution of the data.

## Discussion

### Adaptive DFBA algorithm

Predefined metabolic changes known prior to the simulation can also be implemented using the DOA formalism, but entangle iteratively running successive simulations between concentration change points, i.e. running a separate DOA calculation for each continuous period without nutrient changes. Simulating interactive metabolite changes triggered by unpredictable events with DOA would need to run a full-length calculation, inspect it, detect the occurrence of the first key event, extract concentrations at that time, apply the desired changes, restart the calculation from that point with the new concentrations and calculate the system evolution to completion again, revising the new results to detect any next key event and repeating the cycle as many times as needed.

Our approach does not require calculations to be restarted while permitting on-the-fly introduction of arbitrary changes to the model or to nutrient concentrations.

When changes are known in advance, specifying metabolite changes in static tabular format provides a convenient solution, whereas using a function provides the additional control needed to implement feed-back controls in response to dynamic and unpredictable system changes: by monitoring the levels of all metabolites at each time point, it is possible to immediately detect when trigger points are reached and react accordingly.

Example uses of this functionality include the response of a sensor in a feed-back controlled, fed-batch experiment, the effect of addition of a given substrate in flask cultures at desired time points, or the effect of depletion of nutrients caused by concomitant processes.

More complex schemes are possible modifying the model status at any point. Using a tabular format permits modification of reaction rates when their course is known in advance and can be modelled mathematically, e.g. multi-phase linear models [27], sigmoid fitting [22], or any other. A function provides the additional versatility needed to implement arbitrarily complex changes in metabolic behaviour, like implementing any desired expression for calculating uptake rates (e.g. Michaelis-Menten [9, 11]), setting reaction bounds in response to changes of directly or indirectly related fluxes or nutrient concentrations (e.g. dependence of CO_2_ on pH [22]), implementing concentration-dependent boundary conditions (e.g. phosphate-dependent secretory and metabolic changes [11, 33]), activating specific genes in response to environmental and internal conditions [10, 29], implementing time-dependent behavioural changes (e.g. CO_2_/O_2_ uptake/excretion switches in response to day-night light cycles [22]), or detecting differential trends to act accordingly.

These modifications permit unprecedented arbitrary complexity in steering simulations to model dynamic regulatory, genomic or even epigenomic changes with Adaptive DFBA.

Users may choose FBA or MTF to solve the metabolic problem at each step. We have tested several combinations of ODE solvers and architectures. To summarize, GLPK provides the best cost-benefit trade-off, and if it fails to run on a specific calculation (which should be exceptional), then CLP (which is slower) or CPLEX provide suitable alternatives.

### Overproduction of secretory proteins in *S. lividans*

It has been shown that poor cell growth can be associated with increased secretion of endogenous and heterologous products [3, 34], and that secretion cannot easily be coupled to other objectives [3, 18, 33–35]. Protein secretion poses a well-known additional problem since conflicting objectives lead to a controversial DFBA solution: if priority is given to growth, the cell will limit secretion to save resources, whereas if priority is given to secretion, it will proceed at the expense of growth. Various approaches to deal with these conflicts have been proposed with varying success such as assuming that secretion is tied to cell growth [12], to glucose uptake [18] or using alternate objectives, such as ATP production [17].

We have shown several examples of DFBA limitations when applied to secretory protein overproduction and how to overcome those using Adaptive DFBA, reporting the simplest models that still gave good results in each case. More complex parameter-tuning and pre-processing (e.g. growth estimation using sigmoid functions or rate approximations using non-linear functions) can be easily implemented in R. Using Adaptive DFBA we could enforce specific rates such as growth, L-alanine, C-source or protein secretion and explore which limits are compatible with observed experimental behaviour.

Even with minimal intervention in the system constraints and a coarse time step, different cellular responses and their metabolic associations could be identified using Adaptive DFBA: harbouring the pIJ486 multicopy plasmid or overexpressing secretory proteins resulted in changes to the dynamic consumption of various nutrients in a delicate balance. As in previous metabolomics studies [36], both the plasmid-bearing and mTNF-α producing strains diverge from the plasmid-free strain, with the mTNF-α overproducing strain becoming more divergent with time.

*S. lividans* showed an initial preference for using amino acids as the main C and N sources, resulting in an initial increase in NH_4_^+^ levels and sustained saccharide concentration, coincident with previous observation in batch and fed-batch cultures [27, 28]. As amino acids levels decreased, glucose/mannitol and NH_4_^+^ became preferential sources of C and N, and L-alanine was excreted to the medium in large quantities [36]. This switch was associated with a temporary alteration of growth rate when the change was too abrupt. Once the switch completed, growth resumed its former speed until glucose/mannitol reduction forced a new switch to L-alanine consumption. This reduced the need for glucose/mannitol and NH_4_^+^ and was associated to the switch to the stationary phase (often near the crossover point between glucose/mannitol and L-alanine levels). These data also yield useful information to interpret previous observations of Tat- or Sec-dependent secretory protein overproduction using glucose or mannitol using *S. lividans* TK21 [3, 28, 29]. Interestingly, DFBA calculations without enforcement of proper L-alanine exchange rates were also viable, although they proved unable to survive for as long as simulations where it was controlled. This suggests that L-alanine excretion may not be required in early growth, which, may better fit an immediate optimal FBA solution, and that its excretion might be a suboptimal metabolic mechanism leading to constitution of an external reservoir that can sustain viability after the main nutrients are depleted, and explaining the need for forced controls using Adaptive DFBA.

The size and composition of the overproduced secretory protein affects metabolic patterns: interestingly, heterologous proteins like mouse mTNF-α and *Rhodothermus* cellulase-A have a larger, size dependent, metabolic footprint and lead to faster amino acid uptake than overexpression of α-amylase from *S. lividans* or agarase from the closely related *S. coelicolor*, suggesting mutual adaptation of metabolism and protein composition in *S. lividans*.

### Analysis of time-dependent relationships

The availability of a versatile method to model complex systems paves the way for exploring time-dependent interactions. FBA-based studies consider a single point in time, associations are heavily dependent on the magnitude of fluxes and their differences and necessarily ignore time-dependent changes.

Having access to dynamic simulation data opens the possibility of exploring time-related associations, such as identifying fluxes or metabolites that display a highly correlated, anti-correlated or independent time behaviour, clustering reactions by flux patterns, grouping and ranking them functionally and investigating associations to specific targets.

Our results suggest that using only roughly approximate limits for key reactions may be enough to obtain acceptable predictive models. While these limits may be provided by educated guesses, proper modelling should rely on statistically sound multivariate models, as no single metabolite could be completely associated to growth or protein secretion. Selecting an appropriate metabolite combination becomes then the most important issue. All three methods gave relatively consistent results, however, in our hands, Boruta’s Random Tree based approach seems better suited than traditional feature extraction methods to identify biologically meaningful variables.

Application of feature extraction methods identified relevant associations with H_2_O_2_ (likely as an indicator of oxidative stress, which is associated to the onset of the stationary phase, to the decline in α-amylase secretion and to the surge in agarase production), L-alanine, mannitol, other amino acids and, surprisingly, four metallic ions (Mo^2+^, Ni^2+^, Cu^2+^, Co^2+^) whose association with secretion had never been noticed before. Coincidentally, a recent study [34] reported that *S. lividans* grown in minimal medium (MM) had a low yield in mRFP protein secretion, whereas growth in CM/glucose had a high yield, on the opposite side of the spectrum, only paralleled by NB medium (a rich medium with peptic digest of animal tissue and beef extract). The only qualitative differences between MM and CM/glucose are the presence in CM/glucose of CuSO_4_ and CoCl_2_. Furthermore, that a carefully controlled, minimal medium like CM/glucose, containing only glucose and various ions, can match in efficiency a very rich, complex medium like NB, further supports their key role. While the relevance of these ions has passed unnoticed to date, these experimental findings strongly validate the use of deductions based on methods of feature extraction from simulated calculations.

We used heatmaps to identify metabolites whose time-dependent evolution differed significantly and which could be selected as targets for elaborating roughly predictive time-dependent models for use in the specification of approximate exchange limits. Although these methods may be used to obtain approximate predictions, further work is clearly needed to construct better predictive models. A potentially promising approach should likely consider the dependence of each metabolite on all other relevant ones to build multivariate models.

Adaptive DFBA expands the field of metabolic systems whose time-dependent evolution can now be analysed, extending DFBA applicability to deal with uncorrelated objectives, dynamically adapting and unforeseeable systems, and improving predictive power in situations with reduced experimental data.

Our analyses may certainly be improved. Possible enhancements to Adaptive DFBA include additional fine-tuning, further exploring the effect of other key metabolites identified thanks to these simulations, modelling the role of relevant post-secretory mechanisms such as the effect of protein folding and modification, the role of external proteases in degrading misfolded proteins [37] to account for losses in activity, or implementing reactions for “endogenous metabolism” (digestion of dead cells to reduce biomass and produce constituent monomers for survival) in late growth phases [11]. At the simulation level, while our calculations using relatively large models can be completed in acceptable times, further optimizations might include enabling dynamic modification of the time step size [38] and addition of further extensions to the algorithm such as Robustness Analysis, Phenotypic Phase Plane Analysis, or Minimization of Metabolic Adjustment Analysis, which might improve its utility in biotechnology engineering [18]. As mentioned, there is still room open for improvement of predictive models; whether these are really worth implementing will depend on future research.

## Conclusions

We have shown the advantages of the Adaptive DFBA approach with its descriptive application to reproduce and explore experimental data, and its exploratory utility for modelling *S. lividans* secretion via the major Sec or the minor Tat secretion routes.

Our work not only opens the way for advanced and versatile simulation of arbitrarily complex metabolic systems, but also allows exploration of time-dependent relationships that were not easily accessible until now due to the difficulty in modelling arbitrary systems with DFBA.

## Availability and requirements

**Project name:** AdaptiveDFBA

**Project Home Page:** https://github.com/jrvalverde/AdaptiveDFBA

**Archived version:** 08-Feb-2019 DOI:10.5281/zenodo.2560170

**Operating System:** Platform Independent

**Programming Language:** R

**Other requirements:** an ODE solver (one or more of GLPK, CLP, LP-SOLVE or CPLEX) and its R binding, R Sybil package

**License:** GPL/EU-GPL

**Any restrictions to use by non-academics:** None

## Supplementary information

**Additional file 1: Table S1.** Sample commands to reproduce the calculations. **Figure S1.** Simulation of wild-type *S. lividans* TK24. **Figure S2.** Simulation of *S. lividans* TK24 pIJ486. **Figure S3.** Simulation of *S. lividans* TK24 pIJ486 overproducing Sec-secreted mTNF-α. **Figure S4.** Simulation of overproduction of Tat-secreted agarase by *S. lividans* TK21 pIJ486. **Figure S5.** Simulation of overproduction of Sec-secreted α-amylase by *S. lividans* TK21 pIJ486. **Figure S6.** Simulation of *S. lividans* TK24 pIJ486 overproducing Sec-secreted cellulase-A. **Figure S7.** Sample correlations between amino acid and agarase exchanges. **Figure S8**. Sample correlations between amino acid and amylase exchanges. **Figure S9.** Variable importance computed after Boruta’s method. **Figure S10.** Partial heatmap of Mann-Whitney’s U *P*-values. **Figure S11.** Examples of quadratic regression fits. (DOCX 5348 kb)

## Data Availability

The datasets supporting the conclusions of this article are available in the GitHub repository https://github.com/jrvalverde/AdaptiveDFBA Project Name: AdaptiveDFBA. Project Home Page: https://github.com/jrvalverde/AdaptiveDFBA Archived version: 2019-02-15. DOI:10.5281/zenodo.2560170 Operating System: Platform Independent. Programming Language: R. Other requirements: an ODE solver (one or more of GLPK, CLP, LP-SOLVE or CPLEX) and its R binding, R Sybil package. License: GPL/EU-GPL.

## Abbreviations

ATP: Adenosine triphosphate
C: Carbon
DFBA: Dynamic Flux Balance Analysis
DNA: Deoxyribonucleic acid
DOA: Dynamic Optimization Approach
FBA: Flux Balance Analysis
FVA: Flux Variability Analysis
GSMN: Genome-Scale Metabolic Network
L-ala: L-alanine
MTF: Minimization of the Total Flux
mTNF-α: mouse Tumor Necrosis Factor α
N: nitrogen
ODE: Ordinary Differential Equation(s)
PMF: Proton Motive Force
*S. coelicolor*: *Streptomyces coelicolor*
*S. lividans*: *Streptomyces lividans*
*S.lividans*-TK21-AmlB: *Streptomyces lividans* TK21 pIJ486 overexpressing α-amylase encoded by the *S. lividans amlB* gene
*S.lividans*-TK21-DagA: *Streptomyces lividans* TK21 pIJ486 overexpressing gen *dagA* encoding agarase from *Streptomyces coelicolor*
*S.lividans-*TK21-pIJ486: *Streptomyces lividans* TK21 harbouring the multicopy plasmid pIJ486
*S.lividans*-TK24-CelA: *Streptomyces lividans* TK24 pIJ486 overexpressing the *Rhodothermus marinus* thermostable cellullase-A gene *celA*
*S.lividans*-TK24-mTNFα: *Streptomyces lividans* TK244 pIJ486 overproducing Sec-secreted mTNF-α
*S.lividans-*TK24-pIJ486: *Streptomyces lividans* TK24 harbouring the multi-copy plasmid pIJ486
SOA: Static Optimization Approach
