# Supplementary material for: Dynamic metabolic modelling of overproduced protein secretion in Streptomyces lividans using adaptive DFBA
Source: BMC Microbiol. 2019 Oct 26;19:233. doi: 10.1186/s12866-019-1591-7 (PMC6815373; doi:10.1186/s12866-019-1591-7)
Supplement: Supplementary file 1 — Additional file 1: Table S1. Sample commands to reproduce the calculations. Figure S1. Simulation of wild-type S. lividans TK24. Figure S2. Simulation of S. lividans TK24 pIJ486. Figure S3. Simulation of S. lividans TK24 pIJ486 overproducing Sec-secreted mTNF-α. Figure S4. Simulation of overproduction of Tat-secreted agarase by S. lividans TK21 pIJ486. Figure S5. Simulation of overproduction of Sec-secreted α-amylase by S. lividans TK21 pIJ486. Figure S6. Simulation of S. lividans TK24 pIJ486 overproducing Sec-secreted cellulase-A. Figure S7. Sample correlations between amino acid and agarase exchanges. Figure S8. Sample correlations between amino acid and amylase exchanges. Figure S9. Variable importance computed after Boruta’s method. Figure S10. Partial heatmap of Mann-Whitney’s U P-values. Figure S11. Examples of quadratic regression fits. (DOCX 5348 kb) [file 12866_2019_1591_MOESM1_ESM.docx]

Additional file 1

**Figure S1: Simulation of wild-type *S. lividans* TK24**

| A  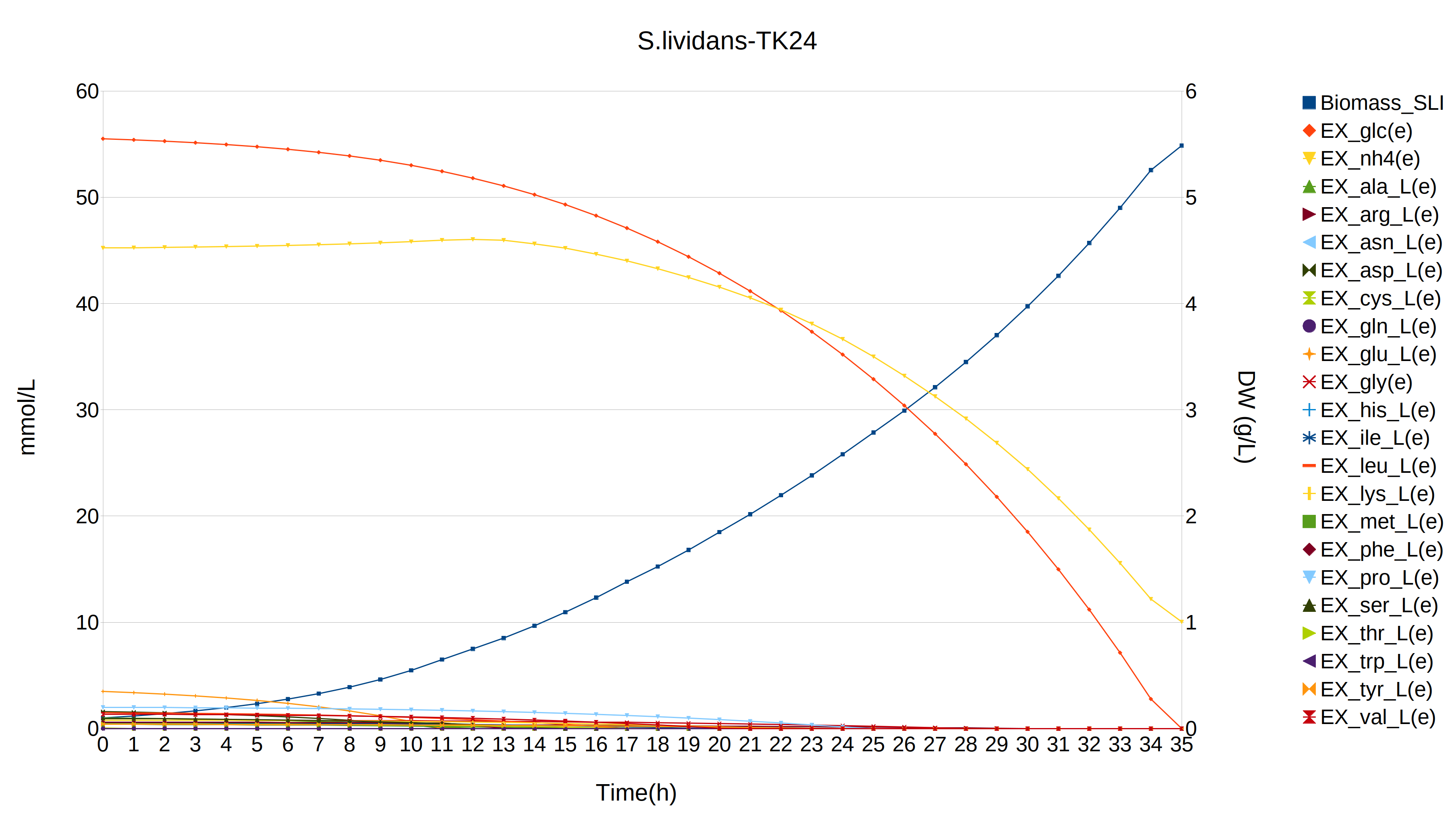 |
| --- |
| B  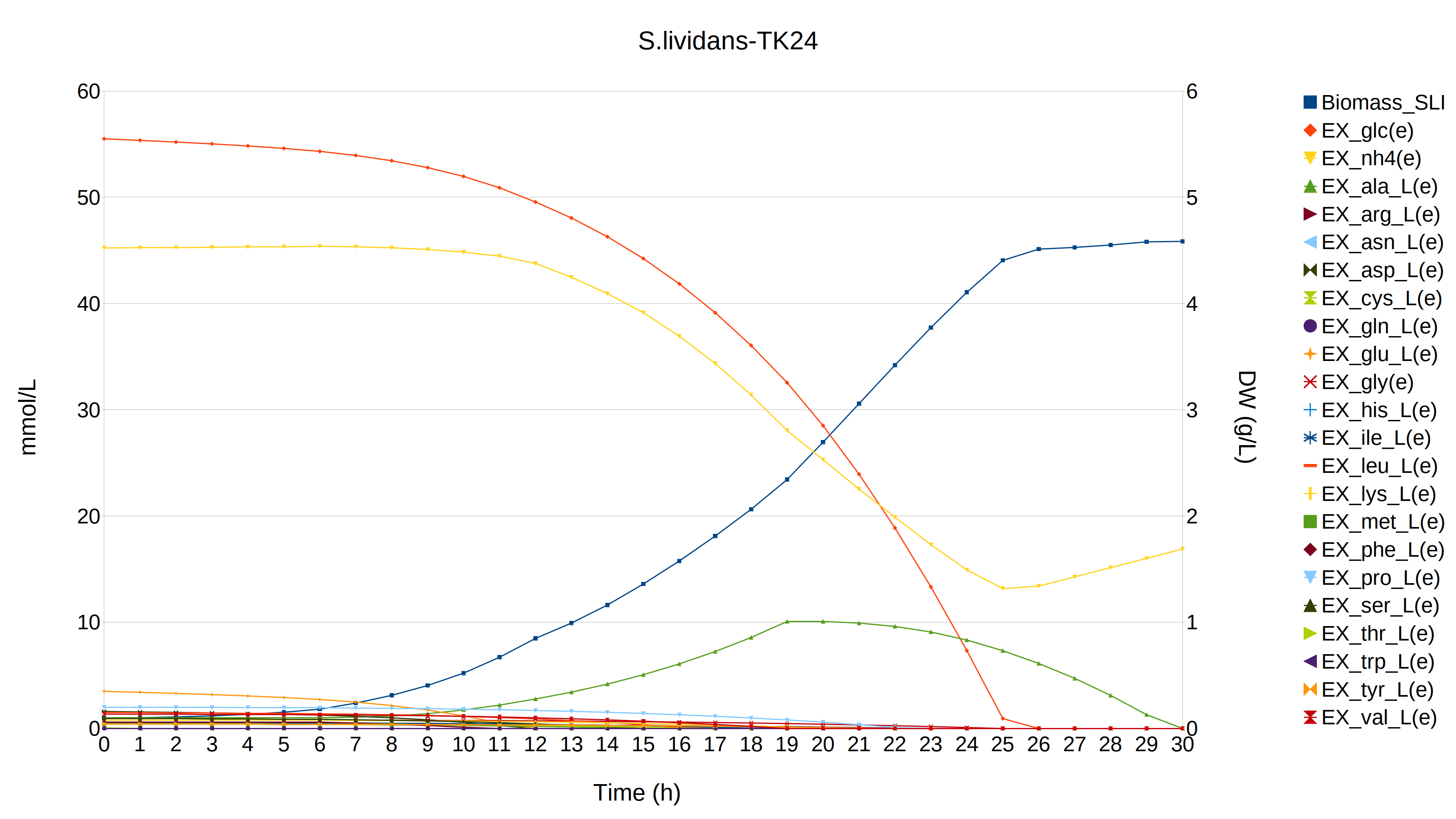 |

A (up): results of a classical DFBA simulation. B (down): result of the Adaptive DFBA simulation. For each simulation, two line plots are produced: one representing Biomass (g/L) vs. time (h) and a second one representing metabolite concentration (mmol/L) vs. time (h) for selected metabolites. Differences in growth and usage of glucose (red), NH_4_^+^ (yellow), L-alanine (green) and Biomass (blue) are easily appreciable.

**Figure S2: Simulation of *S. lividans* TK24 pIJ486**

| A  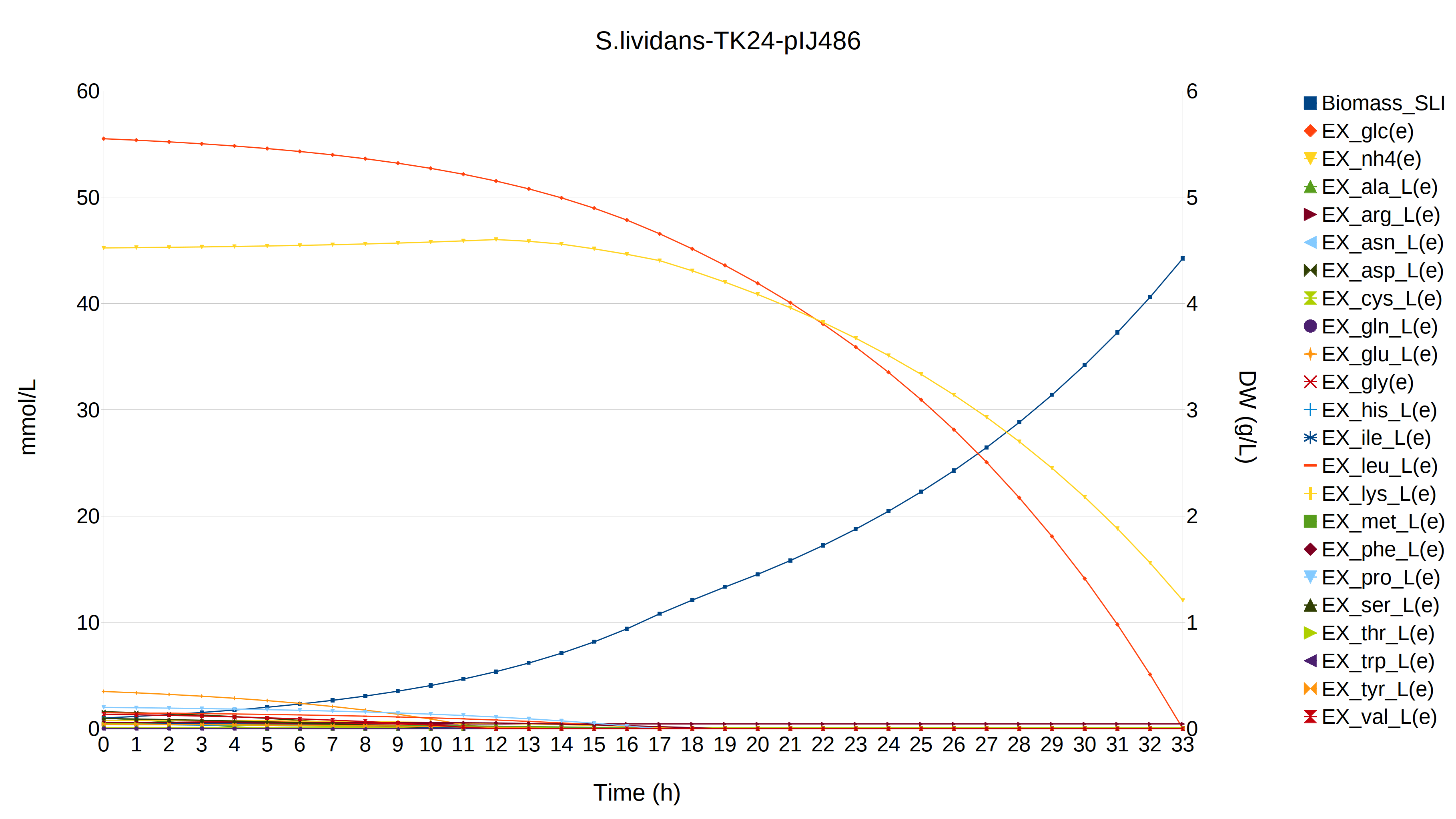 |
| --- |
| B  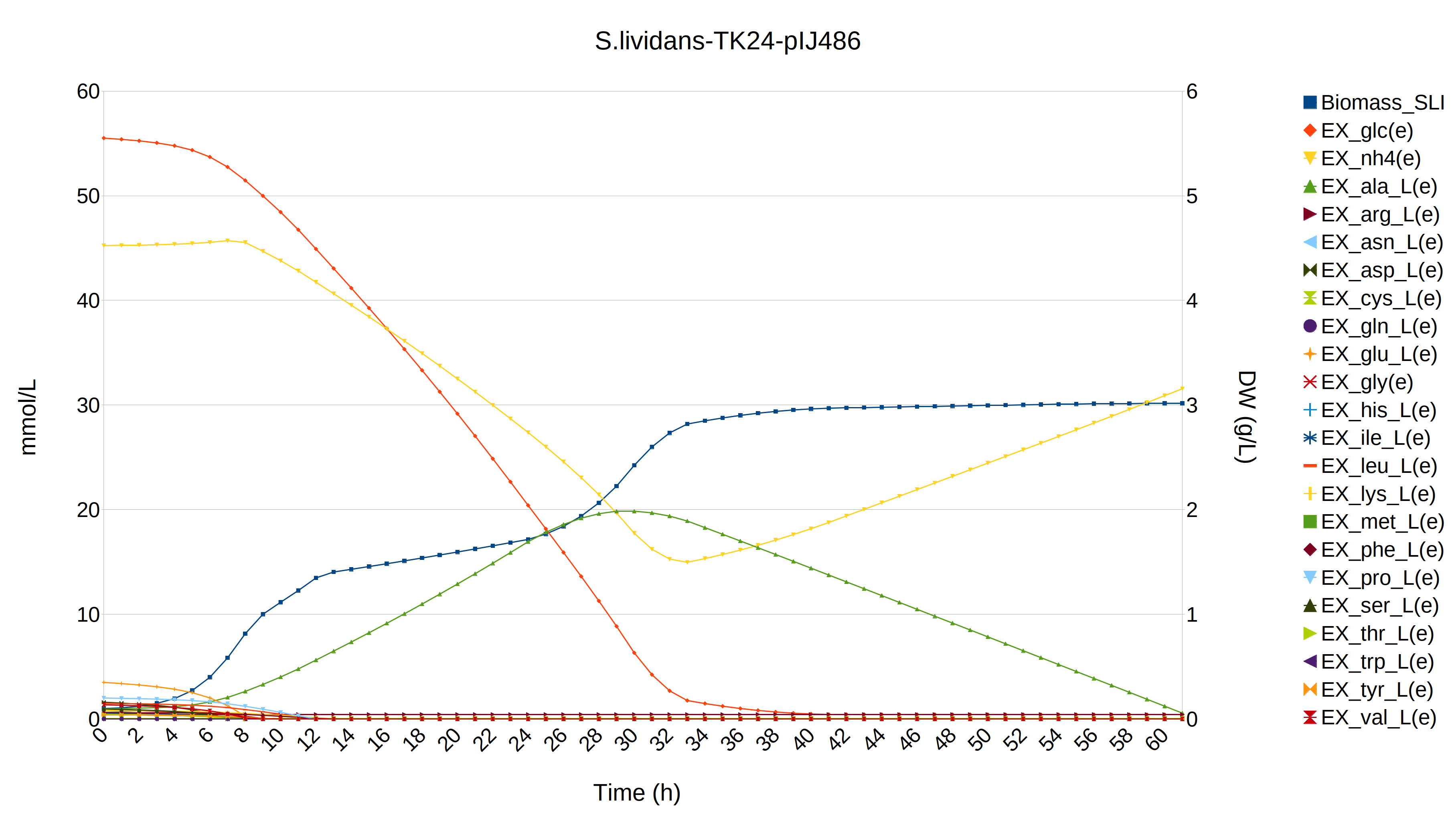 |

A: DFBA (up) and B: Adaptive DFBA (down). For each simulation, two line plots are produced: one representing Biomass (g/L) vs. time (h) and a second one representing metabolite concentration (mmol/L) vs. time (h) for selected metabolites.

**Figure S3: Simulation of *S. lividans* TK24 pIJ486 overproducing Sec-secreted mTNF-α**

| A  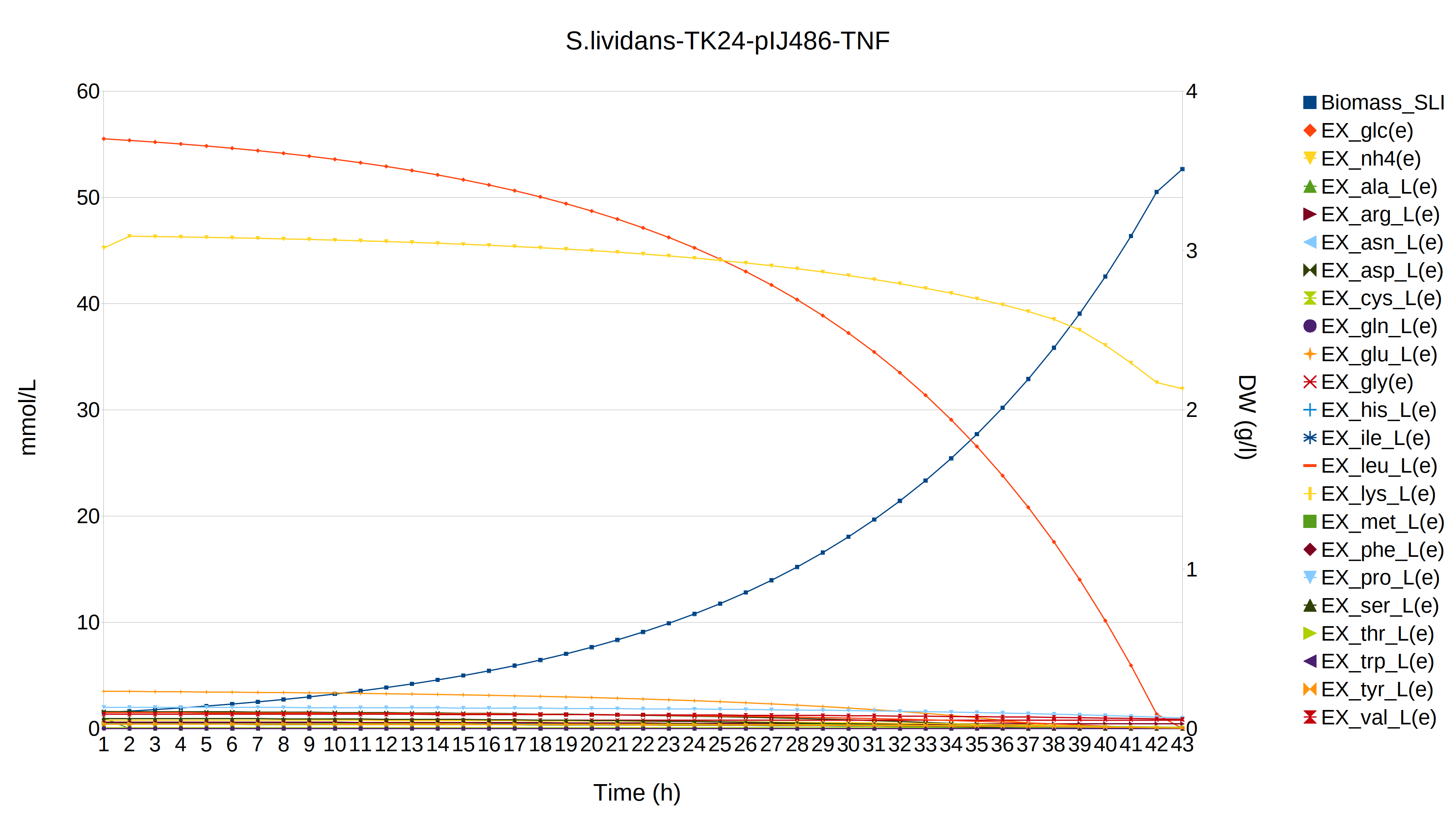 |
| --- |
| B  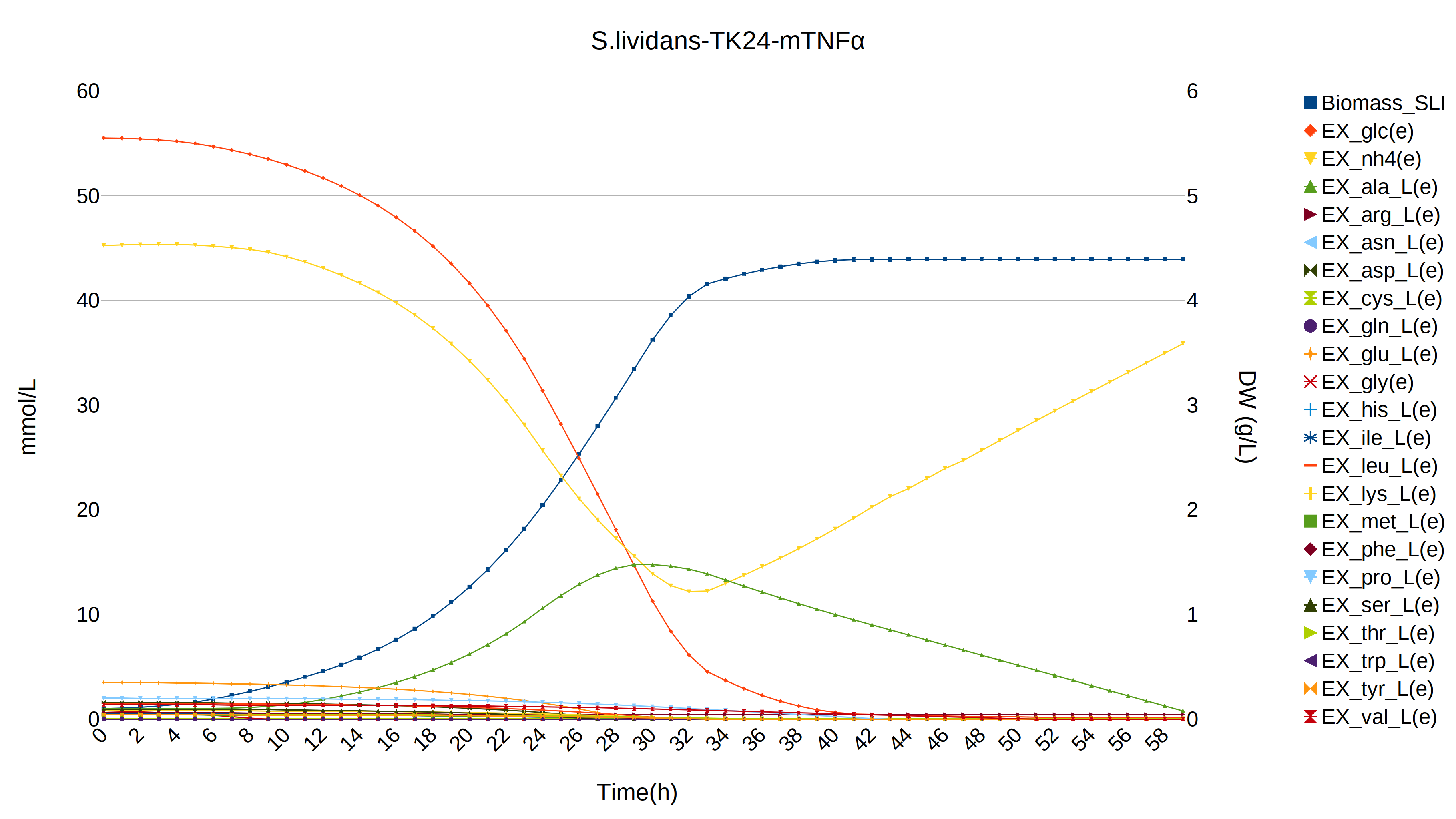 |

A) DFBA (up) and B) Adaptive DFBA (down). For each simulation, two line plots are produced: one representing Biomass (g/L) vs. time (h) and a second one representing metabolite concentration (mmol/L) vs. time (h) for selected metabolites.

**Figure S4: Simulation of overproduction of Tat-secreted agarase by** ***S. lividans* TK21 pIJ486**

| A  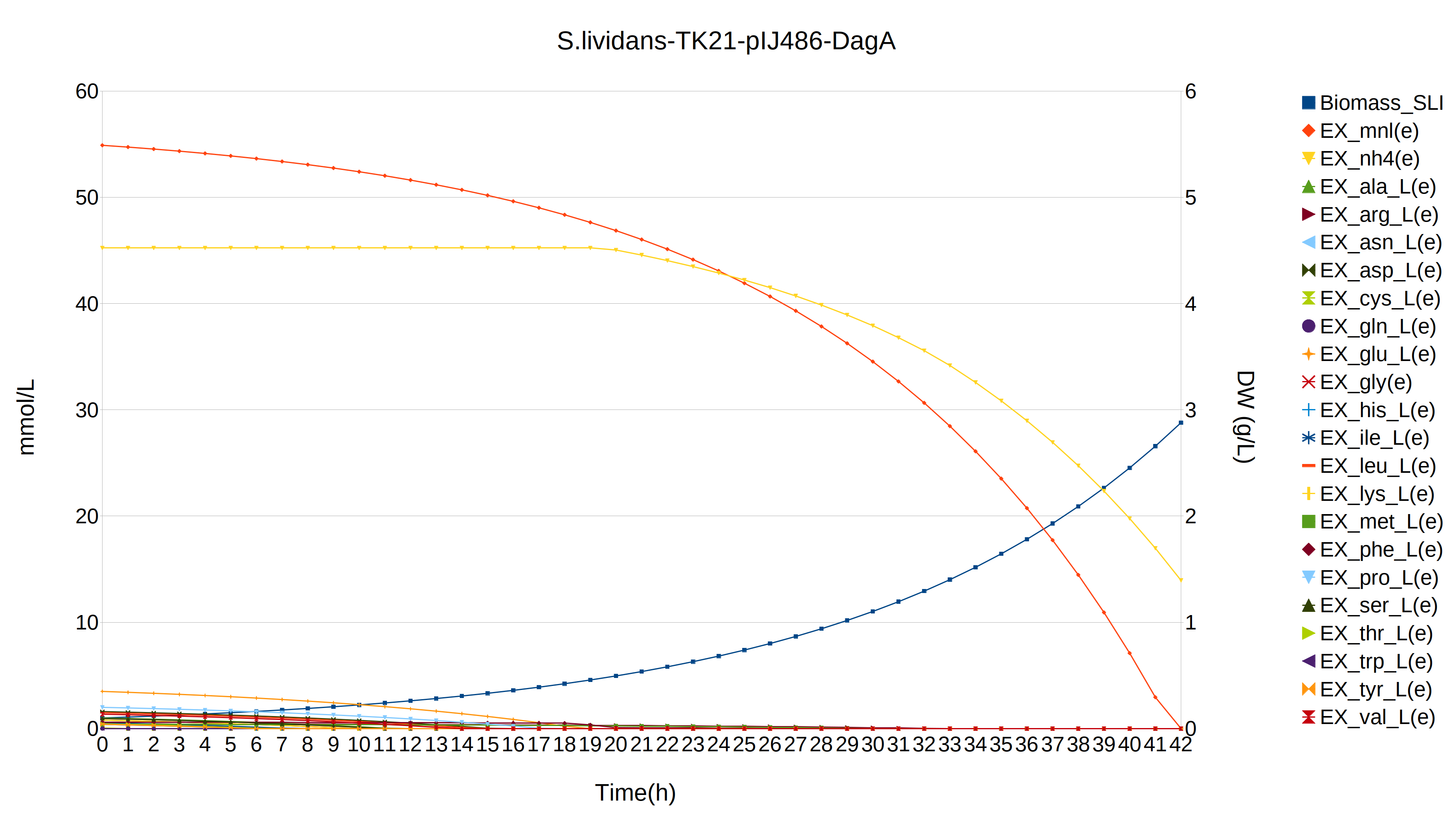 |
| --- |
| B  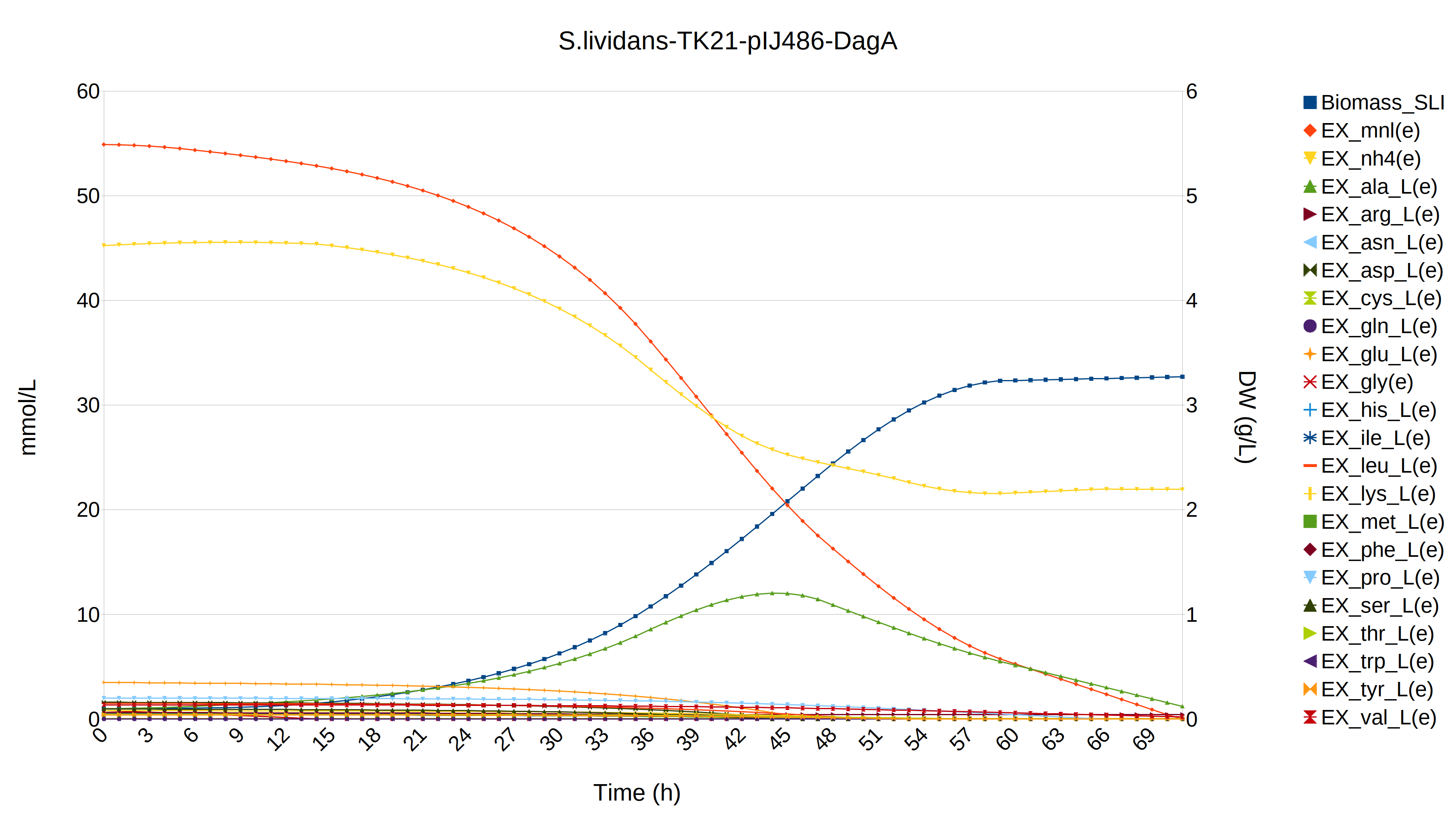 |

A) Up: DFBA results. B) Down: Adaptive DFBA results. For each simulation, two line plots are produced: one representing Biomass (g/L) vs. time (h) and a second one representing metabolite concentration (mmol/L) vs. time (h) for selected metabolites (mannitol, NH_4_^+^ and amino acids).

**Figure S5: Simulation of overproduction of Sec-secreted α-amylase by *S. lividans* TK21 pIJ486**

| A  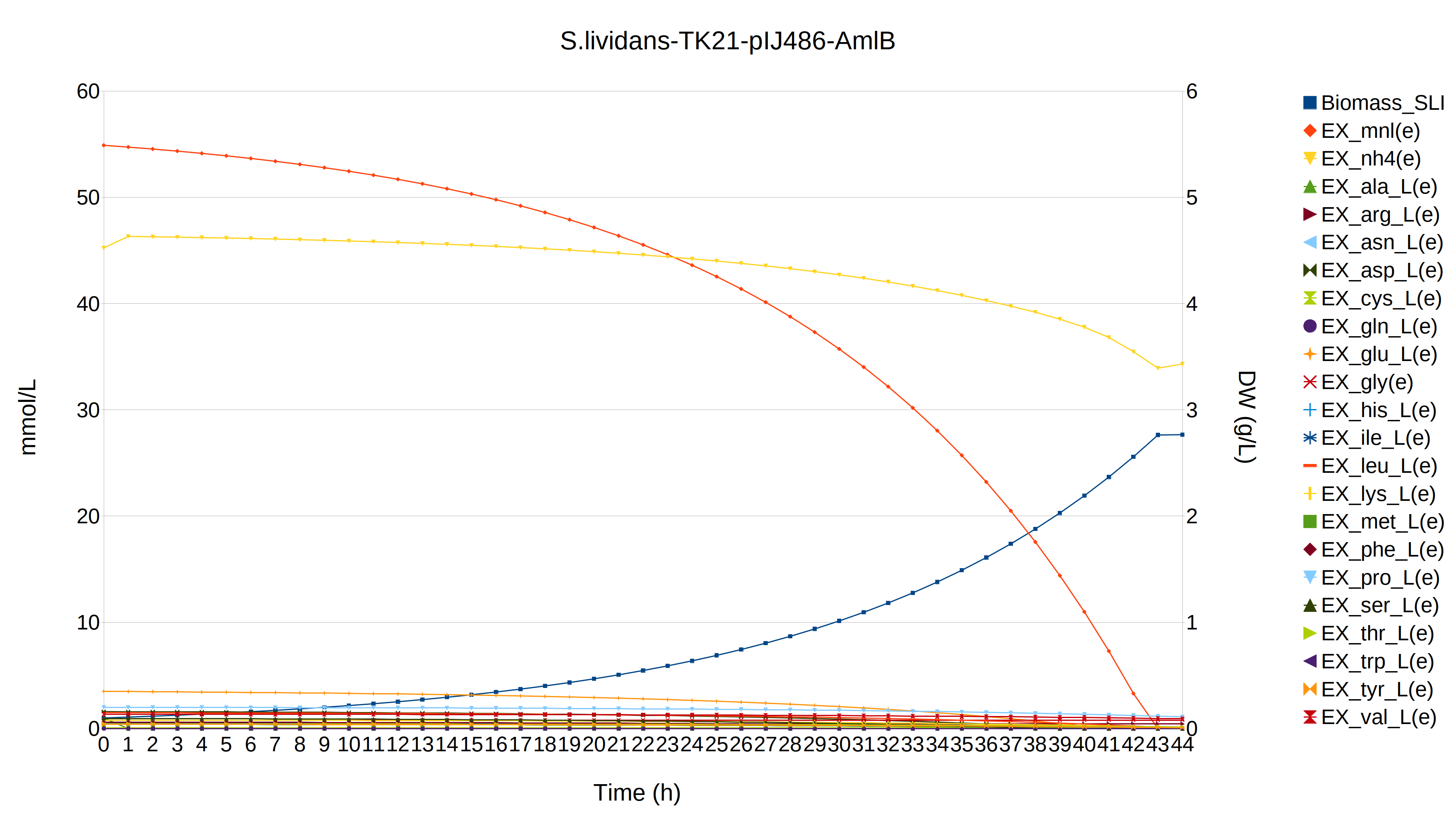 |
| --- |
| B  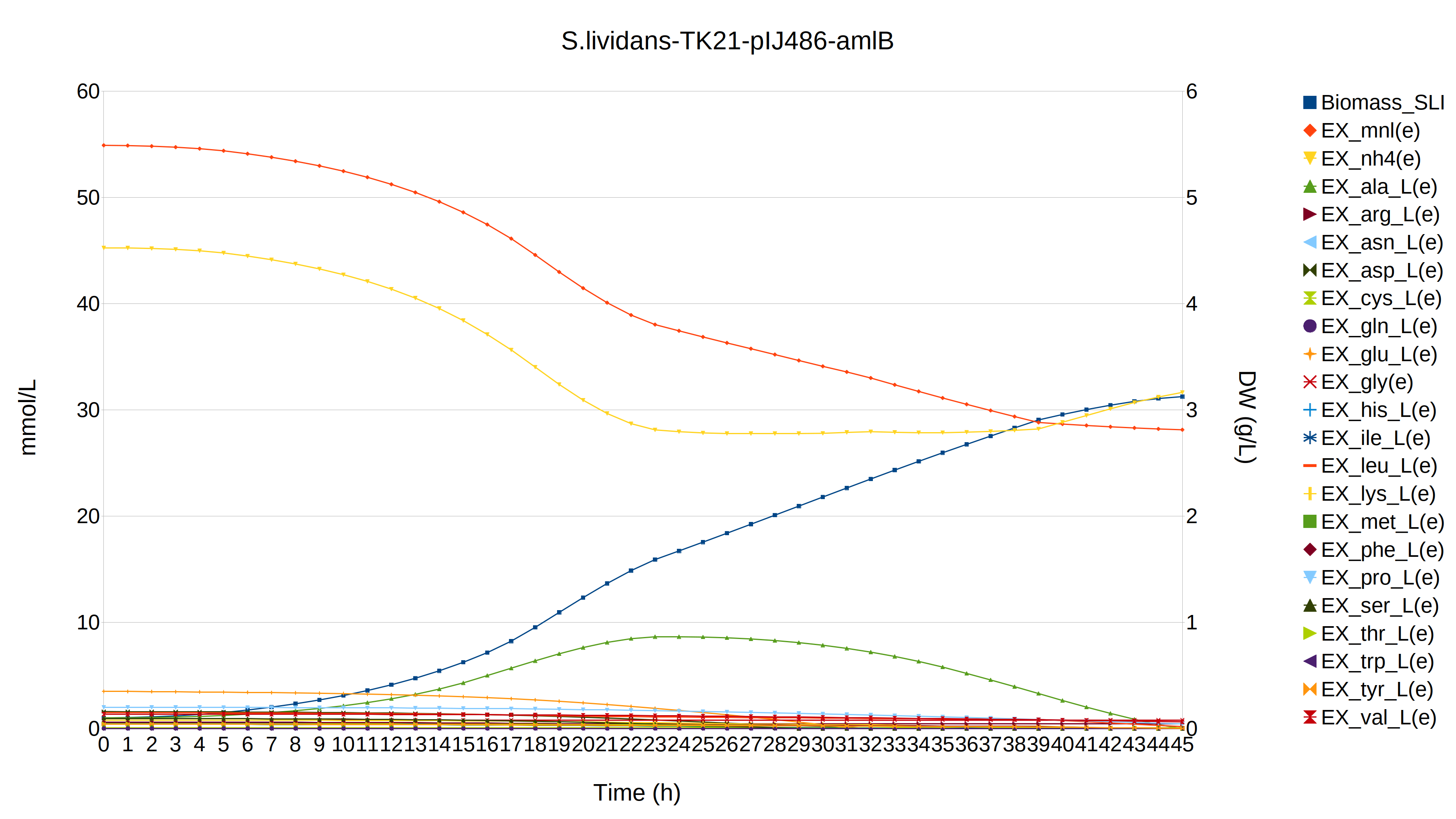 |

A) Up: DFBA. B) down: Adaptive DFBA. For each simulation, two line plots are produced: one representing Biomass (g/L) vs. time (h) and a second one representing metabolite concentration (mmol/L) vs. time (h) for selected metabolites (mannitol, NH_4_^+^ and amino acids).

1. **Figure S6: simulation of *S. lividans* TK24 pIJ486 overproducing Sec-secreted** **c****ellulase-A**

| A  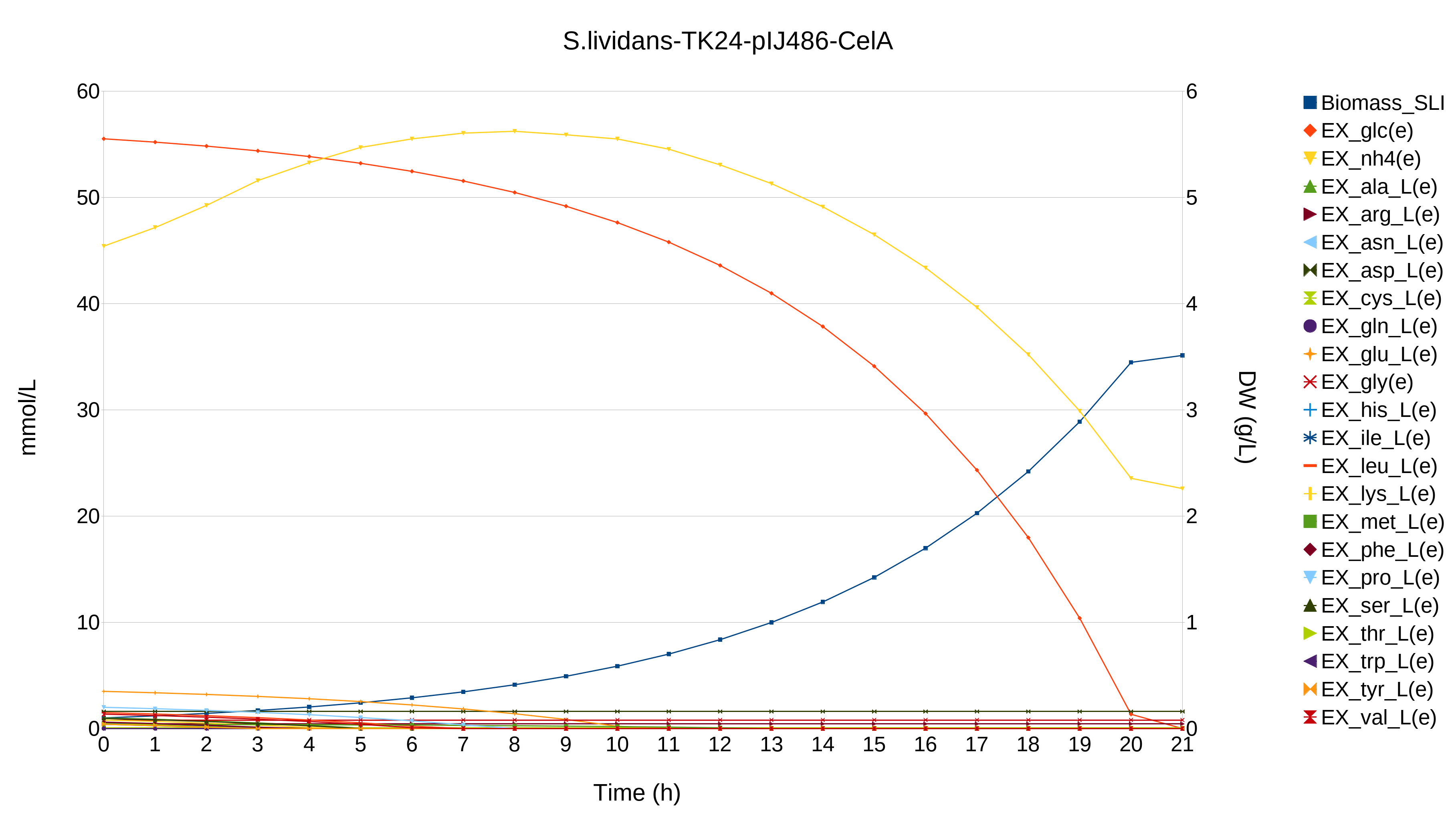 |
| --- |
| B  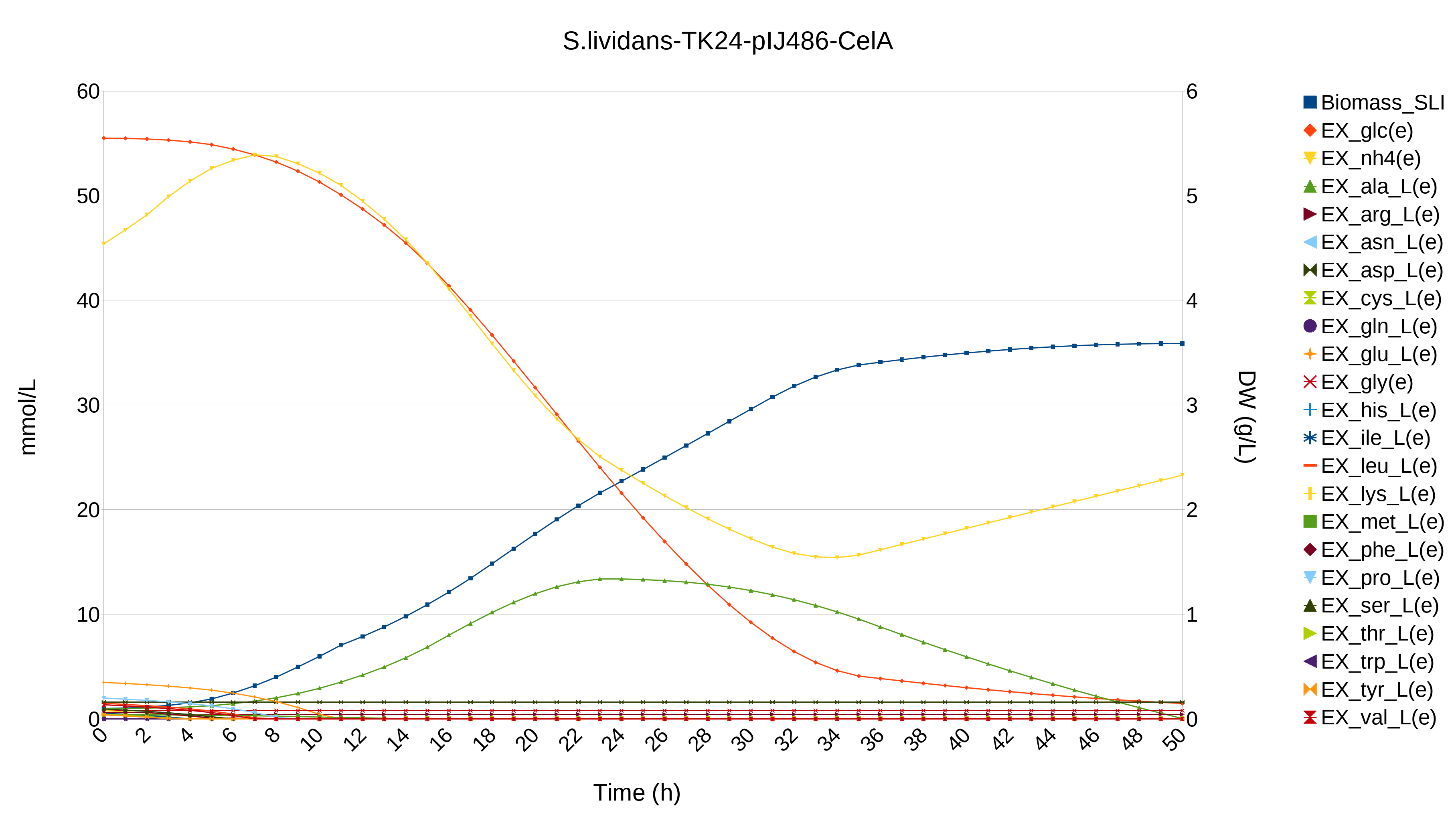 |

1. **A) Up**: DFBA, **B)** down: Adaptive DFBA. For each simulation, two line plots are produced: one representing Biomass (g/L) vs. time (h) and a second one representing metabolite concentration (mmol/L) vs. time (h) for selected metabolites.

# Reproducing the results

Since data preparation for use in such a versatile algorithm can be very difficult to reproduce given the large variety of approaches possible, we have reported the simplest models that still gave good results in each case. More complex parameter-tuning and pre-processing (e.g. growth estimation using sigmoid functions, rate approximations using non-linear functions, Michaelis-Menten function-dependent exchanges, responsive changes, etc..) can be easily implemented in R.

All the data used for the Adaptive DFBA calculations is provided as R datasets in the distributed package and can be easily reproduced following the sample recipe included in the help page for the implemented method (Table S1). Note that, due to R namespace conventions, R should be restarted for each sample model to avoid mixing simulation parameters.

**Table S1:** Sample commands to reproduce the calculations.

| # You’ll need some packages first:  #install.packages(‘sybil’, dep=T)  # and an ODE solver (GLPK, CLP, LP-SOLVE or CPLEX) and its R binding  # NOTE: Gurobi may be installed with a binding available from Sybil  # authors on request.  # e.g. after installing the corresponding ODE solver(s)  #install.packages(c(‘glpkAPI’, ‘cplexAPI’, ‘clpAPI’, ‘lpSolveAPI’), dep=T)  #  # you will need to install the package the first time  # download it from GitHub, extract and install with ‘devtools’  #library(devtools)  #install(‘adfba’, dep=T)  library(adfba)  help(adaptiveDFBA) # to see how to use Adaptive DFBA  # load an example data set (E. coli)  data(Ec_core)  lowbnd(Ec_core)[react_id(Ec_core)=='EX_glc(e)'] = -10;  lowbnd(Ec_core)[react_id(Ec_core)=='EX_o2(e)'] = -18;  ## run adaptiveDFBA(), Ec_df will be an object of class  ## optsol_dynamicFBA. This run is equivalent to sybilDynamicFBA::dynamicFBA  Ec_df <- adaptiveDFBA(Ec_core,  substrateRxns={'EX_glc(e)'}, initConcentrations=10,  initBiomass=.035, timeStep=.25, nSteps=17,  verboseMode=3)    ## plot biomass and reactions  plot(Ec_df,plotRxns=c('EX_glc(e)','EX_ac(e)'));  ## Load prepared data for wild-type S. lividans  data(SlividansWT) # other examples are SlividansDAG, SlividansAML,  # SlividansTNF, SlividansCEL or Slividans_pIJ486  ## run adaptiveDFBA() specifying additional dynamic constraints  verbose <- 2  af_sol <- adaptiveDFBA(model,  substrateRxns=substrateRxns,  initConcentrations=initConcentrations,  initBiomass = initBiomass,  timeStep = timeStep,  nSteps = nSteps,  exclUptakeRxns = exclUptakeRxns,  retOptSol = TRUE,  fld = TRUE,  biomassRxn = biomassRxn,  dynamicConstraints = dynamicConstraints,  verboseMode = verbose);  plot(af_sol, plotRxns=plotRxns) |
| --- |

Exchange rate parameters in the simulations provided were specified by defining only the values at key time points (e.g. 16h, 24h, 32h, 48h...) and using linear interpolation between them for the intervening time steps. The simulation output, model and, parameters used for a given simulation may be retrieved by restarting R and using a script like the following (Table S2) as a template (modifying the name of the simulation dataset):

| #!/usr/bin/Rscript  library(adfba)  library(sybilSBML)  load('ref/SlividansWT.Rdata') # <<< change this  vebose <- 4  sink('ref/REF')  af_sol <- adaptiveDFBA(model, substrateRxns=substrateRxns,  initConcentrations=initConcentrations,  initBiomass = initBiomass,  timeStep = timeStep,  nSteps = nSteps,  exclUptakeRxns=exclUptakeRxns,  retOptSol=TRUE,  fld=TRUE,  biomassRxn=biomassRxn,  dynamicConstraints=dynamicConstraints,  verboseMode=verbose);  plot(af_sol, plotRxns=plotRxns);  sink()  png('ref/plotADFBA.png', width=750, height=750)  plot(af_sol, plotRxns=plotRxns);  dev.off  saveModel <- function(model, tsv=TRUE, sbml=TRUE, overwrite=TRUE, outDir=".") {  modelName <- model@mod_name  if (outDir == "") { outDir <- "." }  tsvdir <- paste(outDir, "/", "s_tsv/", sep="")  sbmldir <- paste(outDir, "/", "s_sbml/", sep="")  dir.create(tsvdir, showWarnings = FALSE)  dir.create(sbmldir, showWarnings = FALSE)  tsvName <- paste( tsvdir, modelName, sep="" ); # files will be named  # modelName_{desc\|met\|react}.tsv  sbmlName <- paste( sbmldir, modelName, "_L2v1.xml", sep="" );  if (overwrite == FALSE) {  if ((tsv == TRUE)  && (! file.exists(paste(tsvName, '_react.tsv', sep='')))) {  cat('Writing file ', tsvName, '_{desc\|met\|react}.tsv\n')  modelorg2tsv(model,  prefix=tsvName, suffix="tsv",  extMetFlag="b", makeClosedNetwork=TRUE)  }  # and in SBML format  if ((sbml == TRUE) && (! file.exists(sbmlName))) {  cat('Writing file ', sbmlName, '\n')  writeSBML(model, filename=sbmlName, level=2, version=1)  }  }  else {  if (tsv == TRUE) {  cat('Writing file ', tsvName, '_{desc\|met\|react}.tsv\n')  modelorg2tsv(model,  prefix=tsvName, suffix="tsv",  extMetFlag="b", makeClosedNetwork=TRUE)  }  if (sbml == TRUE) {  cat('Writing file ', sbmlName, '\n')  writeSBML(model, filename=sbmlName, level=2, version=1)  }  }  }  write.table(dynamicConstraints, "rates_ref.dat", sep='\t')  print('dynamicConstraints saved to file rates_ref.dat\n')  names(initConcentrations) <- substrateRxns  write.table(initConcentrations, "medium.dat", sep='\t')  print('initConcentrations saved to file medium.dat\n')  saveModel(model)  print('model saved in directories s_sbml (SBML format) and s_tsv (Sybil TSV format)\n')  print(paste('initBiomass:', initBiomass, '\n'))  print(paste('timeStep:', timeStep, '\n'))  print(paste('nSteps:', nSteps, '\n'))  print(paste('biomassRxn:', biomassRxn, '\n')) |
| --- |

# Statistical analyses

## Figure S7 Sample correlations between amino acid and agarase exchanges


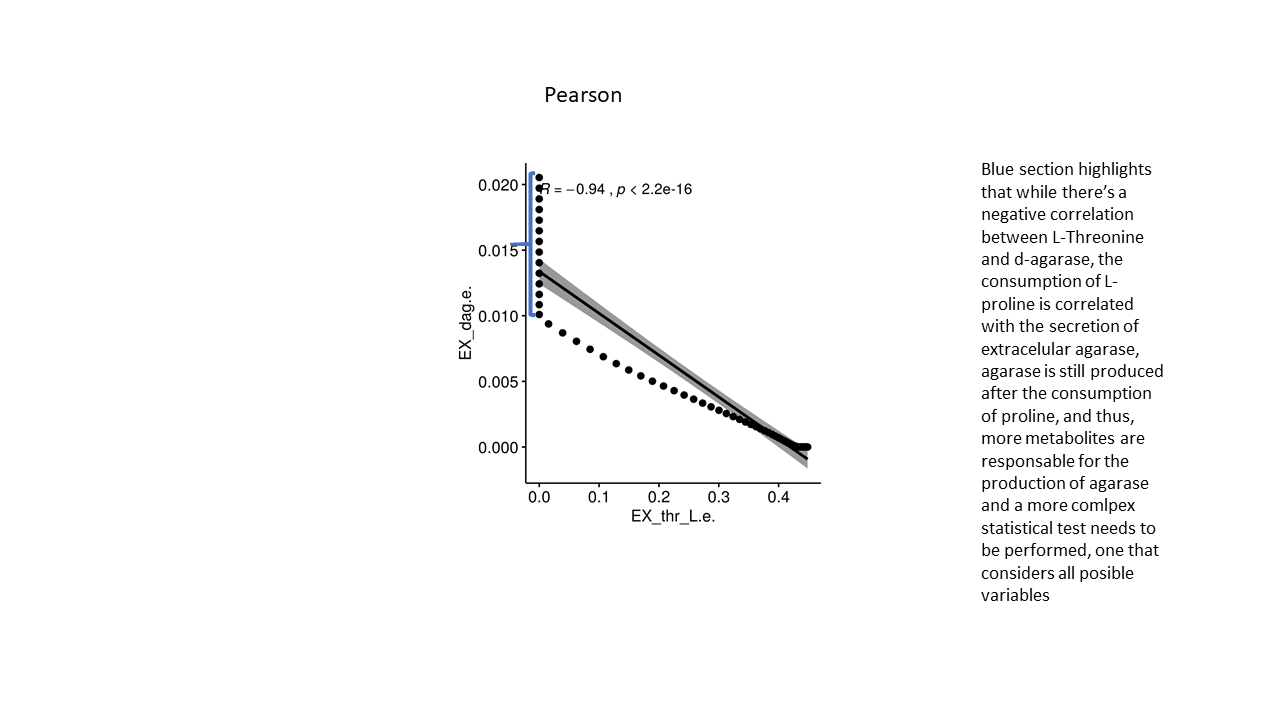

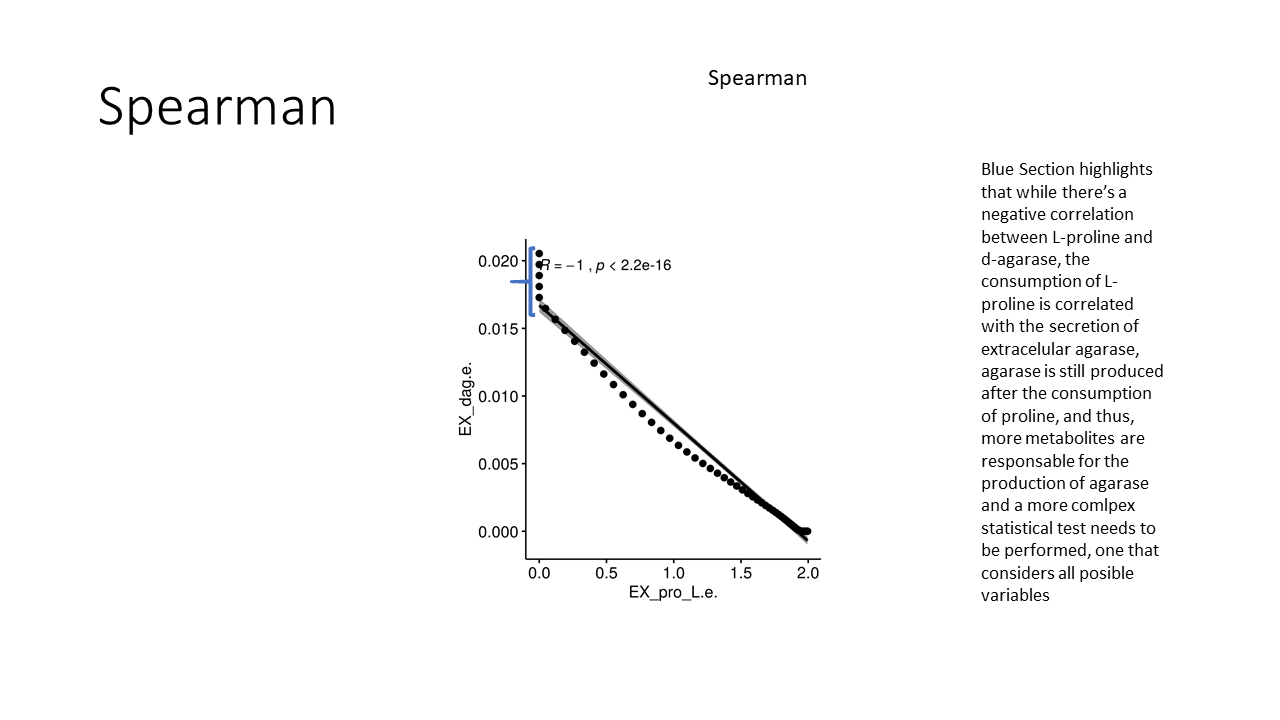


**Correlation between agarase and amino acid exchange.** Left: Pearson correlation of agarase excretion (EX_dag_e) (mmol/L) (Y-axis) and L-Threonine exchange (EX_thr_L_e) (mmol/L) (X-axis). Right: Spearman correlation of agarase concentration (mmol/L) (Y-axis) and L-Proline exchange (EX_pro_L_e) (mmol/L) (X-axis). The blue segment indicates where agarase is being secreted without the consumption of amino acids. A small area of non-correlation may also be appreciated on the right side of each plot (where amino acids are being used, yet no agarase is secreted).

## Figure S8 sample correlations between amino acid and amylase exchanges


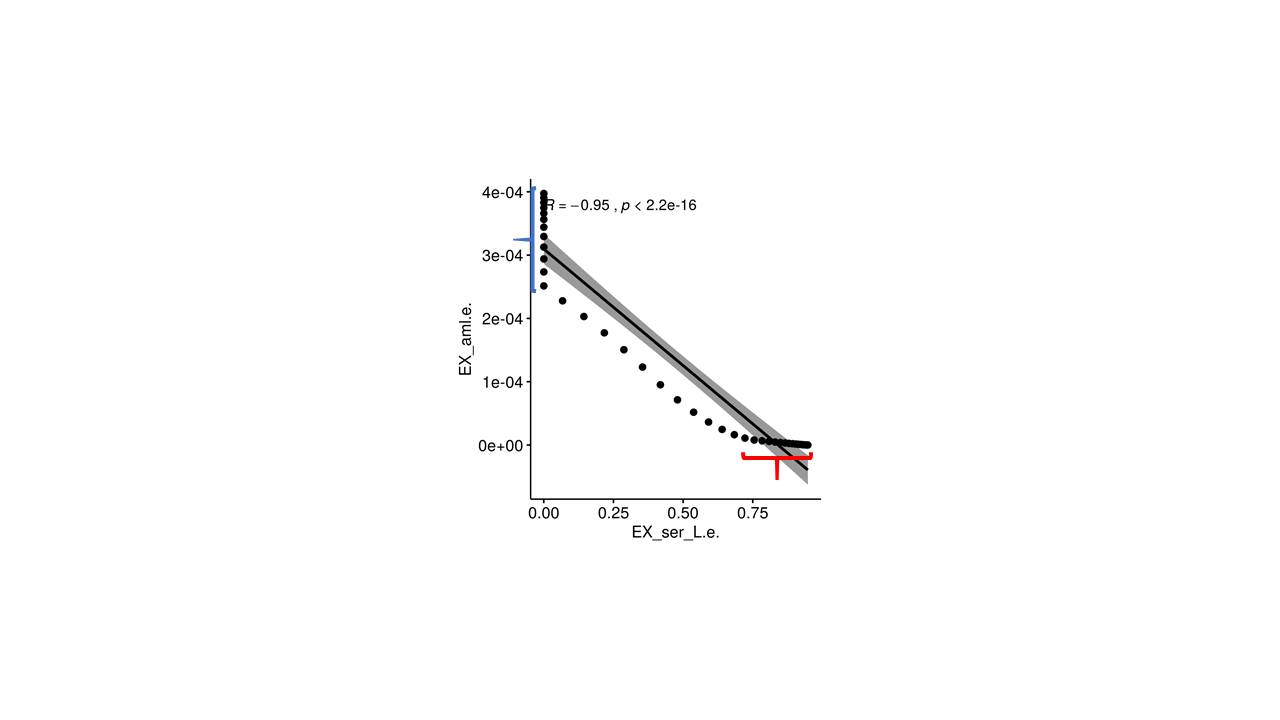

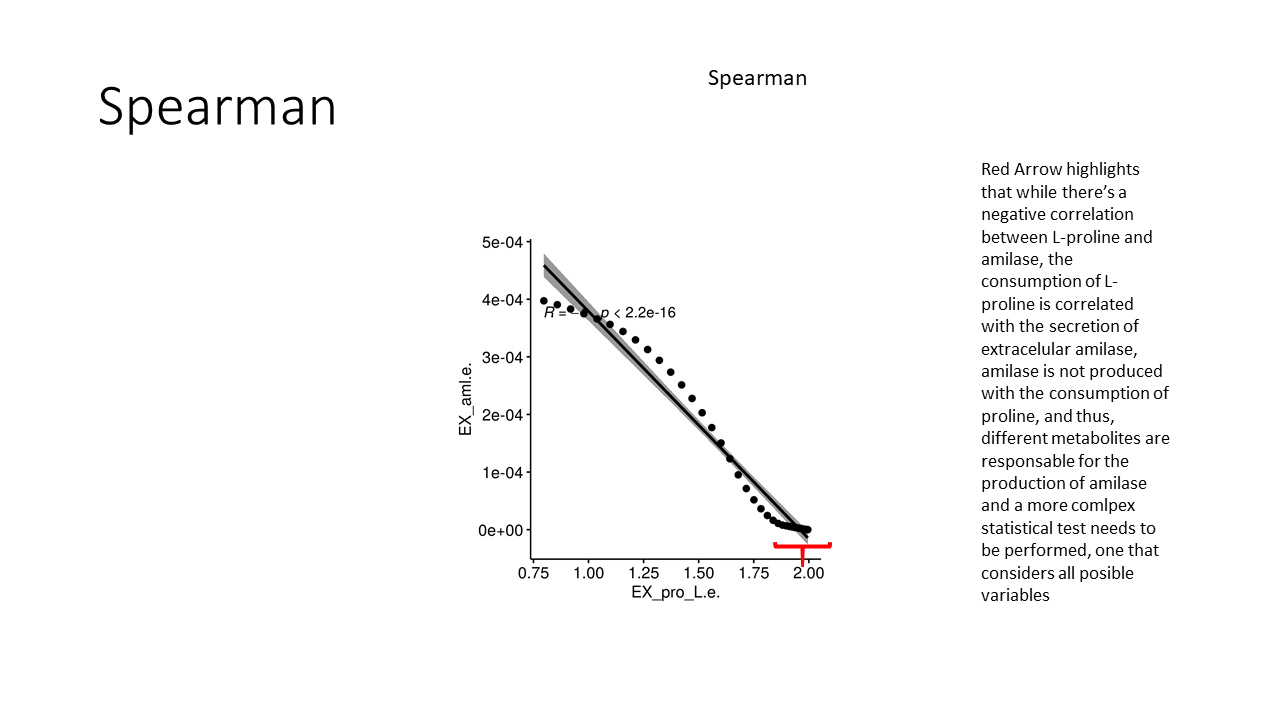


## Correlation between α-amylase and amino acid exchange. Left: Pearson correlation between α-amylase excretion (EX_aml_e) (mmol/L) (Y-axis) and L-Serine exchange (EX_ser_L_e) (mmol/L) (X-axis). Right: Spearman correlation between α-amylase exchange (EX_aml_e) (mmol/L) (Y-axis) and L-Proline exchange (EX_pro_L_e) (mmol/L) (X-axis). The blue segment indicates a region where α-amylase is being secreted without the consumption of amino acids. The red Segment indicates where amino acids are consumed, yet practically no α-amylase is secreted.

## Figure S9: Variable importance computed after Boruta’s method


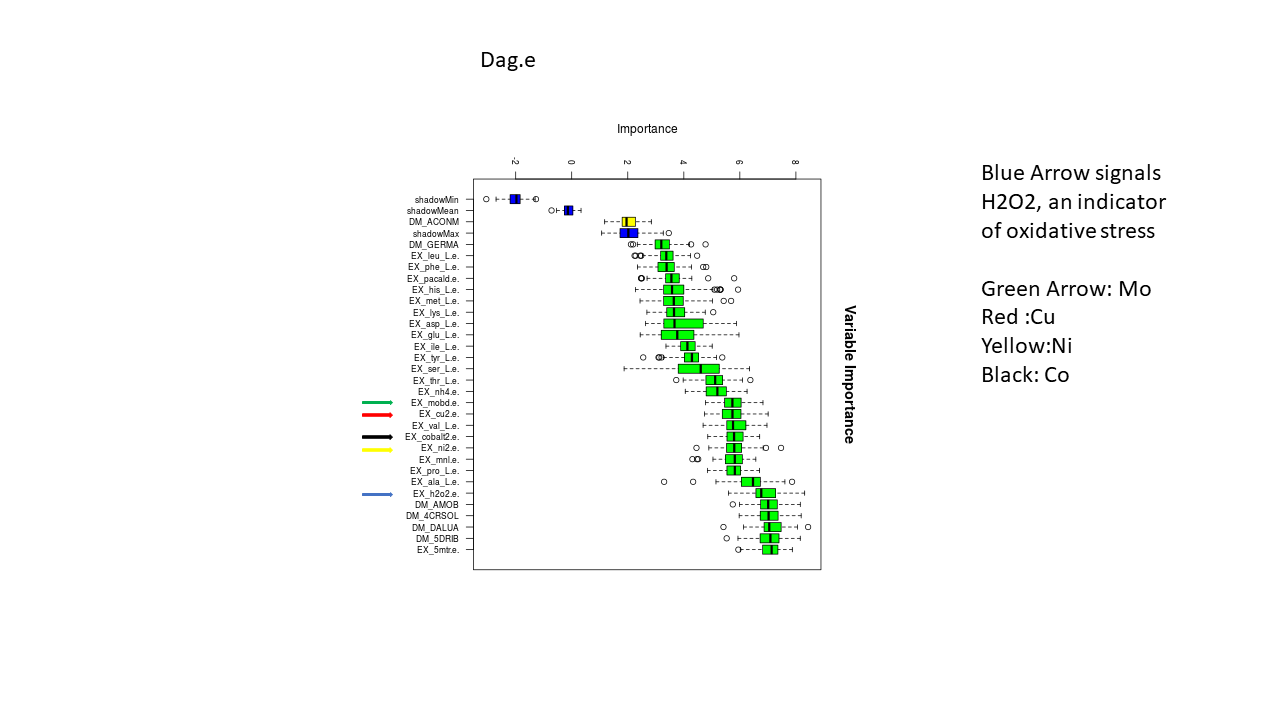

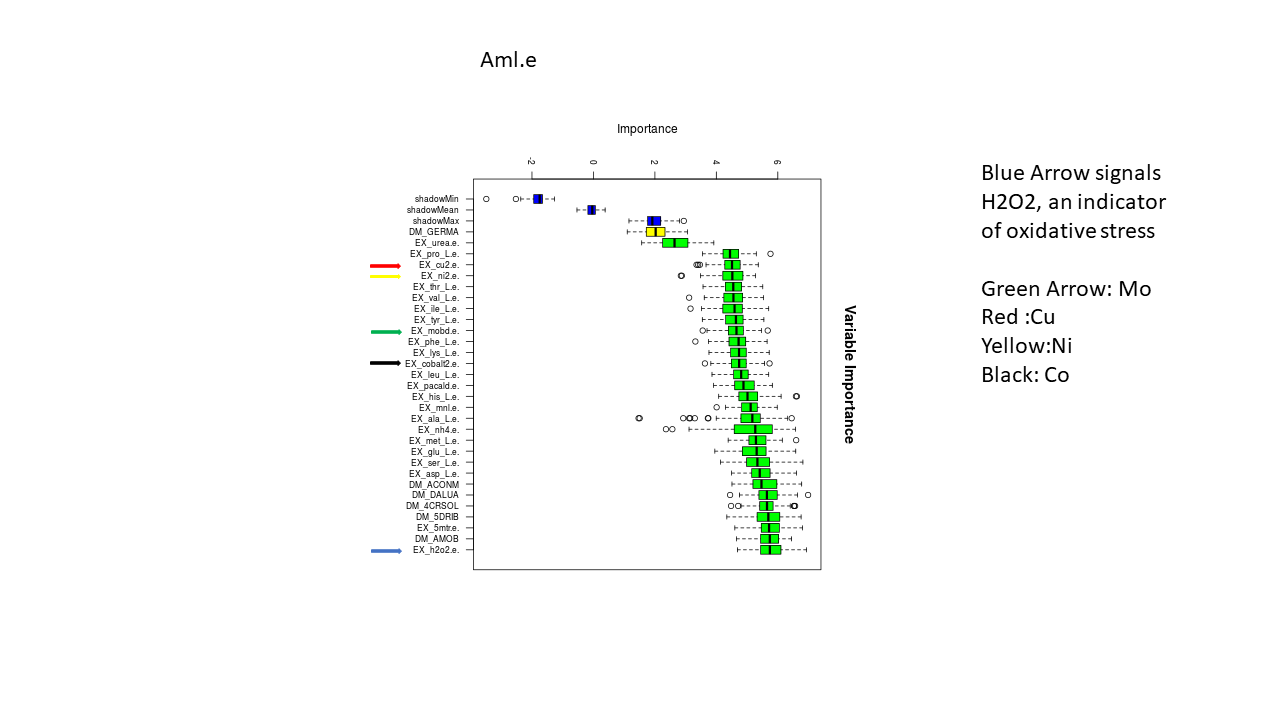


**Variable importance after applying Boruta’s algorithm**. Right: Extracellular agarase secretion. Left: Extracellular α-amylase secretion. X-axis: Metabolites from lower importance to higher. Y-axis: Level of importance. Green box: The metabolite is deemed significantly important. Yellow box: The metabolite might or might not be important. Blue box: Shadow variables. Blue arrow: H_2_O_2_. Green arrow: Molybdenum^2+^. Yellow arrow: Nickel^2+^. Red arrow: Copper^2+^. Black arrow: Cobalt^2+^.

## Figure S10 Partial heatmap of Mann-Whitney’s U P-values


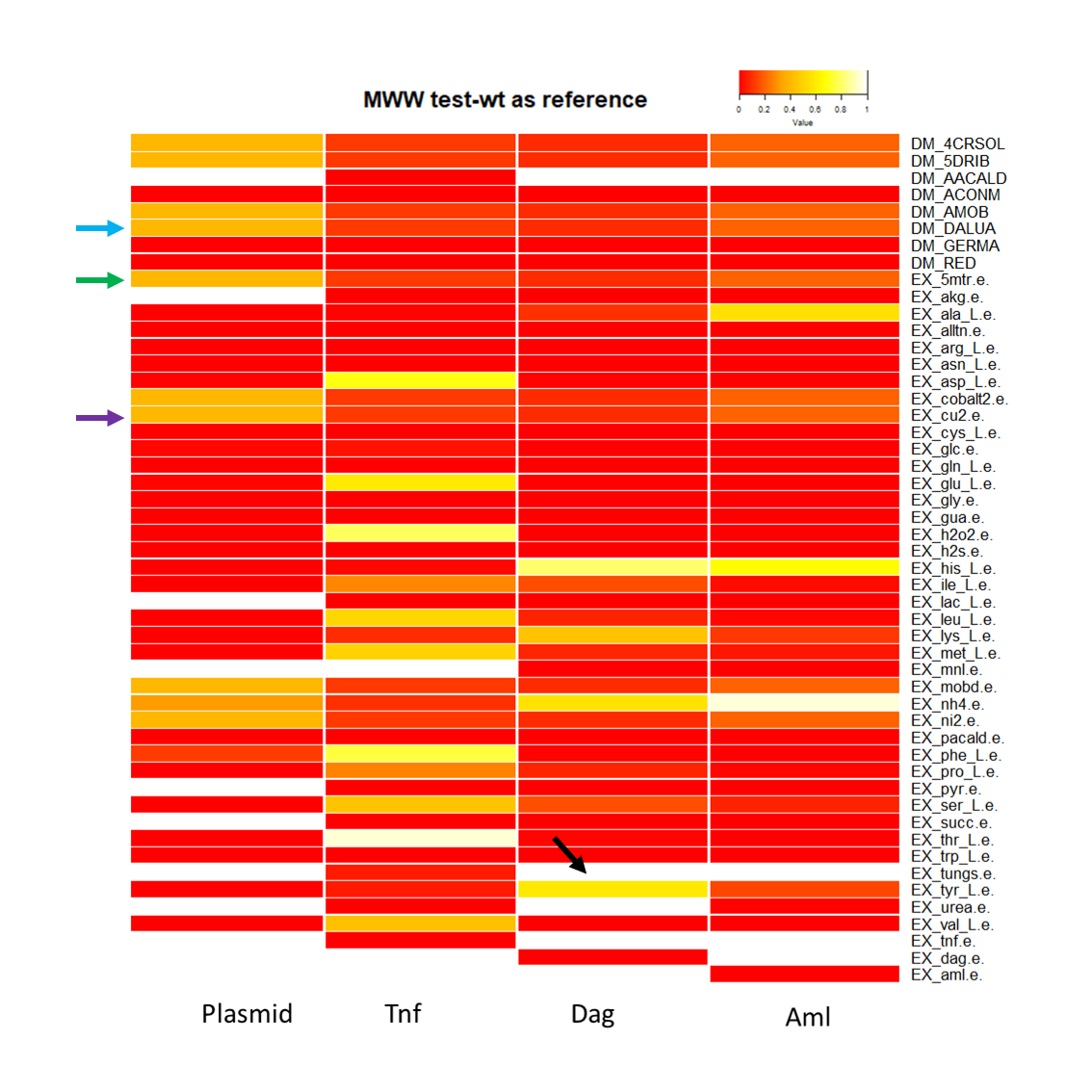


## Partial heatmap of Man-Whitney’s U P-values, using the non-plasmid carrier strain as reference. Each column represents the result of comparing the reference with one of the simulations. Each row corresponds to a different metabolite. White: the test could not be performed for that metabolite on that simulation. Yellow: P-values between 0.4-1. Orange: 0.05-0.4, Red: under 0.05,. Arrows point to sample relationships whose models are reported below: blue arrow: Dialuric acid, green arrow: 5-Methylthio-D-ribose, purple arrow: Cu^2+^, black arrow: L-Tyrosine.

## Figure S11: examples of quadratic regression fits


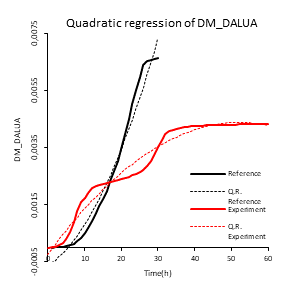

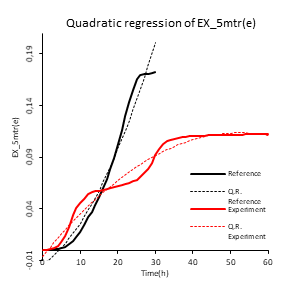


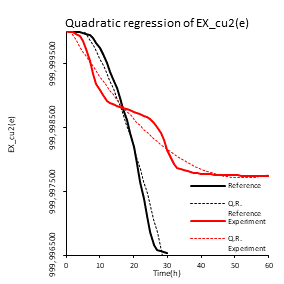

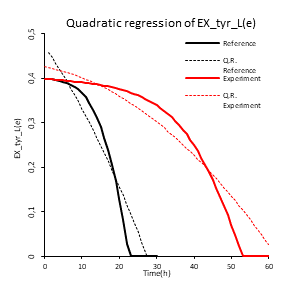


**Comparison of quadratic models.** Solid line: simulation data. Dashed line: values predicted by the Quadratic Regression (Q.R.) model. Upper panel: Non-plasmid carrier *S. lividans* TK24 (reference, black) and *S. lividans* TK24 pIJ486 (experiment, red), left, Dialuric acid concentration (mmol/L) vs. time (hr): right: 5-Methylthio-D-ribose. Lower panel: *S. lividans* TK24 (reference, black) and *S. lividans* TK21 pIJ486 dag (experiment, red), left, Cu^2+^: right, L-tyrosine.
